# Single-Cell RNA Sequencing Analysis of the Immunometabolic Rewiring and Immunopathogenesis of Coronavirus Disease 2019

**DOI:** 10.3389/fimmu.2021.651656

**Published:** 2021-04-14

**Authors:** Furong Qi, Wenbo Zhang, Jialu Huang, Lili Fu, Jinfang Zhao

**Affiliations:** ^1^Institute for Hepatology, National Clinical Research Center for Infectious Disease, Shenzhen Third People’s Hospital, The Second Affiliated Hospital, School of Medicine, Southern University of Science and Technology, Shenzhen, China; ^2^Shenzhen Research Center for Communicable Disease Diagnosis and Treatment of Chinese Academy of Medical Science, Shenzhen, China; ^3^Trinity School of Durham and Chapel Hill, Durham, NC, United States; ^4^Electronic and Computer Engineering, China North Vehicle Research Institute, Beijing, China; ^5^Center for Life Sciences, Tsinghua University, Beijing, China

**Keywords:** COVID-19, metabolic changes, peripheral blood mononuclear cells, inflammation, antibody secretion

## Abstract

Although immune dysfunction is a key feature of coronavirus disease 2019 (COVID-19), the metabolism-related mechanisms remain elusive. Here, by reanalyzing single-cell RNA sequencing data, we delineated metabolic remodeling in peripheral blood mononuclear cells (PBMCs) to elucidate the metabolic mechanisms that may lead to the progression of severe COVID-19. After scoring the metabolism-related biological processes and signaling pathways, we found that mono-CD14^+^ cells expressed higher levels of glycolysis-related genes (*PKM, LDHA* and *PKM*) and PPP*-*related genes (*PGD* and *TKT*) in severe patients than in mild patients. These genes may contribute to the hyperinflammation in mono-CD14^+^ cells of patients with severe COVID-19. The mono-CD16^+^ cell population in COVID-19 patients showed reduced transcription levels of genes related to lysine degradation (*NSD1, KMT2E*, and *SETD2*) and elevated transcription levels of genes involved in OXPHOS (*ATP6V1B2*, *ATP5A1*, *ATP5E*, and *ATP5B*), which may inhibit M2-like polarization. Plasma cells also expressed higher levels of the OXPHOS gene *ATP13A3* in COVID-19 patients, which was positively associated with antibody secretion and survival of PCs. Moreover, enhanced glycolysis or OXPHOS was positively associated with the differentiation of memory B cells into plasmablasts or plasma cells. This study comprehensively investigated the metabolic features of peripheral immune cells and revealed that metabolic changes exacerbated inflammation in monocytes and promoted antibody secretion and cell survival in PCs in COVID-19 patients, especially those with severe disease.

## Highlights

1. COVID-19 patients, especially those with severe cases, showed dramatic metabolic remodeling in immune cells.
2. Enhanced glycolysis and PPP activity may contribute to the hyperinflammation of mono-CD14^+^ cells in severely ill patients.
3. Reduced lysine degradation and OXPHOS in mono-CD16^+^ cells may inhibit M2-like polarization in COVID-19 patients.
4. Increased OXPHOS activity was positively associated with antibody secretion and survival of PCs in patients with severe COVID-19.

## Introduction

Severe acute respiratory syndrome coronavirus-2 (SARS-CoV-2) continues to spread globally, causing widespread morbidity and mortality and showing a tremendously high transmission rate (1). Infection with SARS-CoV-2 is characterized by a broad spectrum of clinical syndromes, which range from asymptomatic disease or mild influenza-like symptoms to severe pneumonia and acute respiratory distress syndrome, often requiring assisted mechanical ventilation and even resulting in death (2, 3). Severe coronavirus disease 2019 (COVID-19) caused by SARS-CoV-2 infection is often associated with older populations and individuals with preexisting conditions, such as cardiovascular disease, diabetes, chronic respiratory disease, and cancer (2).

Recent reports revealed that the progression to severe COVID-19 is associated with immune dysregulation (4). Patients with severe COVID-19 showed drastic changes in their myeloid cell compartments, with an increased proportion of neutrophils and classical (CD14^hi^CD16^lo^) monocytes, dysfunction of HLA-DR^lo^CD163^hi^ and HLA-DR^lo^S100A^hi^CD14^+^ monocytes, and a decreased fraction of nonclassical (CD14^lo^CD16^hi^) monocytes (5). A highly impaired interferon (IFN) response is a hallmark of severe COVID-19 and causes a persistent viral load and immunopathy (6, 7). In patients with severe COVID-19 but not in patients with mild disease, lymphopenia is a common feature, with drastically reduced numbers of CD4^+^ T cells and CD8+ T cells. Lymphopenia or dysfunction of T cells is one of the key indicators of disease progression (8, 9). SARS-CoV-2-specific neutralizing antibodies produced by plasma cells (PCs) are important for viral clearance (10). Critically ill COVID-19 patients but not those with mild symptoms had high concentrations of a fucosylated IgG antibodies against SARS-CoV-2, amplifying proinflammatory cytokine release and acute-phase responses (11). Therefore, antibodies, lymphopenia and inflammatory markers in monocytes may help identify COVID-19 cases and predict their severity.

Metabolism is a fundamental biological process that includes anabolism and catabolism for cell maintenance and growth (12). To date, many studies have focused on the roles of metabolic rewiring in the control of immune responses in various diseases, including COVID-19 (11, 13–15). One such study found that enhanced glycolysis in monocytes and macrophages led to excessive and prolonged production of the cytokines IL-6 and IL-1β in atherosclerosis (15). In COVID-19 patients, enhanced glycolysis in monocytes suppresses the T cell response and promotes epithelial cell death in the lungs (13). Depolarization and dysfunction of mitochondria in monocytes are also correlated with reactive oxygen species generation, proinflammatory cytokine secretion, and cell death in SARS-CoV-2-infected patients (16). Metabolic shifts in CD4^+^ T and CD8^+^ T cells control cell differentiation and inflammation (14, 17). Antibody production is a metabolically demanding process. In patients with HIV infection, antibody glycosylation is determined in an antigen- and pathogen-specific manner, highlighting the importance of metabolic processes in antibody production (18). However, the metabolic alterations related to antibody production in SARS-CoV-2-infected patients are still incompletely understood. Collectively, these findings demonstrate that SARS-CoV-2 infection may cause metabolic alterations in immune cells (subsets of monocytes, T cells and B cells) that contribute to immune dysfunction and disease progression in COVID-19 patients. However, a more in-depth analysis of the metabolic alterations in immune cells and the associations of these alterations with immune cell dysfunction and disease progression in COVID-19 patients remains unclear.

In this study, we reanalyzed single cell RNA sequencing (scRNA-seq) data of peripheral blood mononuclear cell (PBMC) samples from 21 COVID-19 patients (10 with mild and 11 with severe cases) and 11 healthy controls (HCs) to identify the immunometabolic rewiring associated with disease severity. We first assessed and mapped the metabolic landscape of peripheral immune cells from mildly and seriously ill COVID-19 patients by scRNA-seq. We identified several genes of metabolic processes associated with inflammation, antibody production and cell differentiation in these immune cells, providing insight into the metabolic mechanisms underlying disease severity during SARS-CoV-2 infection.

## Materials and Methods

### Sample Collection

The single-cell gene expression data of PBMCs from 21 COVID-19 patients (10 with mild cases and 11 with severe cases) and 11 healthy controls (HCs) were downloaded from the GEO database (https://www.ncbi.nlm.nih.gov/geo/) or GSA database (https://bigd.big.ac.cn/gsa/). The corresponding accession numbers were GSE150728 (19), GSE149689 (20) and HRA000297 (21). Patients older than 70 were excluded. All severely ill patients included in this study needed mechanical ventilation. The clinical characteristics of these patients are listed in **Table S1**. The median days from symptom onset are 14.5 and 11.5 days in mild and severe COVID-19 cases, respectively.

### Single-Cell Filtering, Clustering, Dimensional Reduction, and Visualization

The raw count matrix (UMI counts per gene per cell) was processed with Seurat v3.2.2 (22). Cells with fewer than 200 expressed genes and in which more than 15% of transcripts were mitochondrial genome transcripts were removed. Genes expressed in less than 10 cells were removed. Then, the gene expression data were normalized using the “NormalizeData” function with default settings. The batch-driven sources of cell-cell variation were regressed out using the number of detected UMIs, mitochondrial gene expression data, and ribosome gene expression data, which were implemented using the ‘‘ScaleData’’ function. The corrected expression matrix was used for cell clustering and dimensional reduction. Cell clustering and dimensional reduction were performed using the Seurat package. A total of 2, 000 highly variable genes (HVGs) were selected from the corrected expression matrix and were then centered and scaled using the ‘‘FindVariableGenes’’ function in the Seurat package. Principal component analysis (PCA) was then performed on the HVGs using the ‘‘RunPCA’’ function. The batch effects were removed with the “IntegrateData” function.

Cells were then clustered utilizing the ‘‘FindClusters’’ function by embedding the cells into a graph structure in the PCA space. The parameter resolution was set to 1.5 to identify cell types in all cell populations and in T cell populations. The clustered cells were then projected onto a two-dimensional space using the “RunUMAP” function. The clustering results were visualized with the “DimPlot” function.

### Identifying Various Cell Types

To annotate cell clusters, differentially-expressed genes (DEGs) in each cluster were first identified with the “FindMarkers” function. The cell clusters were then annotated according to a curated set of known cell markers. The cell clusters consistently expressing the same cell marker were merged.

### Scoring of Metabolism-Related Gene Ontology Terms and KEGG Pathways

Metabolism-related Gene Ontology (GO) terms were downloaded from The Gene Ontology Resource (http://geneontology.org/). The child terms of metabolic processes (GO:0008152) were retained (**Table S2**). The metabolism-related Kyoto Encyclopedia of Genes and Genomes (KEGG) terms were downloaded from the KEGG database (https://www.kegg.jp/), and a subset of 48 metabolism-related pathways were extracted for further analysis (**Table S3**). The score of each GO term or KEGG pathway in each cell was calculated using the genes in each term with the AddModuleScore function in Seurat.

### Score Immune Cell Function

Functional signatures of immune cells were calculated using the AddModuleScore function in the Seurat package. The inflammation score of monocytes was calculated using *IFNG, IL10, IL12A, IL13, IL17A, IL18, IL1A, IL1B, IL2, IL21, IL22, IL23A, IL4, IL5, IL6, S100A8, S100A9, S100A10, S100A11, S100A6, S100A12, TNF*, and *CXCL8*. The IFN response score was calculated using *ADAR, APOBEC3, BST2, CD74, MB21D1, DDIT4, DDX58, DDX60, EIF2AK2, GBP1, GBP2, HPSE, IFI44L, IFI6, IFIH1, IFIT1, IRF1, IRF7, ISG15, ISG20, MAP3K14, MOV10, MS4A4A, MX1, MX2, NAMPT, NT5C3, OAS1, OAS2, OAS3, OASL, P2RY6, PHF15, PML, RSAD2, RTP4, SLC15A3, SLC25A28, SSBP3, TREX1, TRIM5, TRIM25, SUN2, ZC3HAV1, IFITM1, IFITM2*, and *IFITM3*. The MHC class II score was calculated using *HLA-DMA, HLA-DMB, HLA-DPA1, HLA-DPB1, HLA-DQA1, HLA-DQB1, HLA-DRA, HLA-DRB1*, and *HLA-DRB5*. The S100 gene family score was calculated using *S100A1, S100A2, S100A3, S100A4, S100A5, S100A6, S100A7, S100A7A, S100A7L2, S100A7P1, S100A7P2, S100A8, S100A9, S100A10, S100A11, S100A12, S100A13, S100A14, S100A15A, S100A16, S100B, S100G, S100P*, and *S100Z*. The aging score was calculated using genes in the GO term aging (GO:0007568). The apoptosis score was calculated using genes in the Apoptosis pathway (hsa04210). The monocyte migration score was calculated using genes in the GO term leukocyte migration (GO:0050900). The T cell migration score was calculated using genes in the GO term T cell migration (GO:0072678). The cytotoxicity score was calculated using *PRF1, IFNG, GNLY, NKG7, GZMB, GZMA, GZMH, KLRK1, KLRB1, KLRD1, CTSW*, and *CST7*. The exhaustion score was calculated using *LAG3, TIGIT, PDCD1, CTLA4, HAVCR2*, and *TOX*. The B cell differentiation score was calculated using genes in the GO term B cell differentiation. The B cell chemotaxis score was calculated using genes in the GO term B cell chemotaxis (GO:0035754). The B cell activation score was calculated using genes in the GO term B cell activation (GO:0042113), and the B cell proliferation score was calculated using genes in the GO term B cell proliferation (GO:0042100).

### Correlation Analysis Between Metabolism-Related Pathways and Immune Function

The correlation between KEGG metabolism-related pathways and immune function in each cell type was evaluated using the scores calculated above with the corr.test function in R (v4.0.2).

### Differential Gene Expression and Gene Coexpression Analyses

The “FindMarkers” function in Seurat with the MAST algorithm (v1.15.0) was used to analyze DEGs. For each pairwise comparison, the “FindMarkers” function was run with the parameter test.use=‘MAST’. A gene was considered significantly upregulated if the average natural logarithm of the fold change (logFC) was > 0.25 and the adjusted *P* was < 0.01. Genes with logFC < −0.25 and adjusted *P* < 0.01 were considered significantly downregulated. Using these DEGs, coexpressed genes were identified using the corr.test function in R. ClusterProfiler (23) in R was used to perform GO term enrichment analysis for the significantly upregulated and downregulated genes. Only GO term of Biological Process was displayed.

## Results

### Changes in the Metabolic Profiles of Immune Cells in COVID-19 Patients

The severity of COVID-19 was categorized as mild, moderate, severe, or critical according to the “Diagnosis and Treatment Protocol of COVID-19 (the 7^th^ Tentative Version)” by the National Health Commission of China. In this study, we grouped patients with mild and moderate COVID-19 into the mild group and assigned those with severe and critical diseases to the severe group. A total of 32 peripheral blood samples—from ten patients with mild COVID-19, eleven patients with severe COVID-19, and eleven HCs—were integrated (19–21). The median days from symptom onset are 14.5 and 11.5 in mild and severe COVID-19, respectively. The demographics and clinical features of these individuals are shown in **Table S1**.

**Figure 1:**
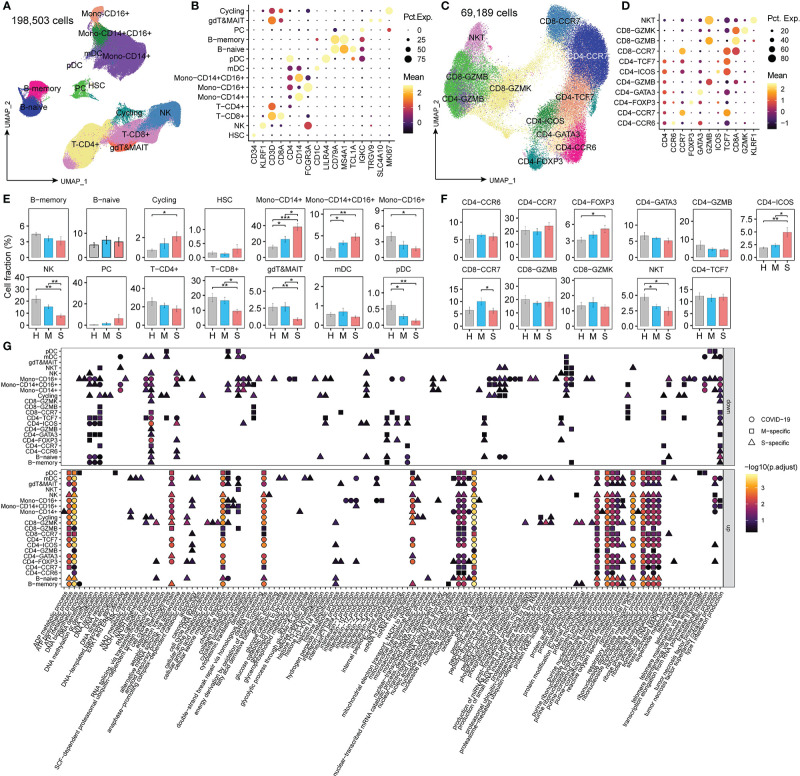
The changed metabolic processes in PBMCs from COVID-19 patients. **(A, C)** Clustering of PBMCs among all cells and T cells, respectively, in COVID-19 patients; **(B, D)** Canonical markers for cell cluster annotation. “Mean” indicates the average gene expression levels. “Pct.Exp.” indicates the percentage of cells expressing the corresponding genes. **(E, F)** The proportion of each cluster in COVID-19 patients. Student’s t-test; *P < 0.05; **P < 0.01; ***P < 0.001. **(G)** The significantly enriched child terms of metabolic processes (GO:0008152) in each cell type using genes differentially expressed in patients with mild or severe disease compared to healthy controls (MAST algorithm; P <0.01, logFC > 0.25 or logFC < -0.25). M-specific: the unique GO terms enriched with DEGs found in patients with mild disease compared to healthy controls. S-specific: the unique GO terms enriched with DEGs found in patients with severe disease compared to healthy controls. COVID-19: GO terms enriched with DEGs found in both mild and patients with severe disease compared to healthy controls.

Using graph-based clustering with uniform manifold approximation and projection (UMAP), a total of 198,503 single cells were reanalyzed and clustered into 14 lineages: mono-CD14^+^ cells (CD14^+^; classical monocytes), mono-CD16^+^ cells (CD16^+^; nonclassical monocytes), mono-CD14^+^CD16^+^ cells (CD14^+^ and CD16^+^; intermediate monocytes), proliferative cells (MKI67^+^; cycling), plasma cells (IGKC^hi^; PCs), B-memory cells (MS4A1^+^), B-naïve cells (TCL1A^+^), plasmacytoid dendritic cells (LILRA4^+^; pDCs), myeloid DCs (CD1C^+^; mDCs), NK cells (KLRF1^+^), γδT & mucosal-associated invariant T (MAIT) cells (TRGV9^+^ and SLC4A10^+^), hematopoietic stem cells (CD34^+^; HSCs), T-CD4^+^ cells (CD3D^+^ and CD4^+^), and T-CD8^+^ cells (CD3D^+^ and CD8A^+^) (**Figures 1A, B**). Then, a total of 69, 189 T cells (CD3D^+^) were further identified as NKT (KLRF1^+^), CD8-CCR7 (naïve), CD8-GZMK (central memory), CD8-GZMB (cytotoxic), CD4-TCF7 (central memory), CD4-ICOS (T follicular help, Tfh), CD4-GZMB (cytotoxic), CD4-GATA3 (Th2), CD4-FOXP3 (Treg), CD4-CCR7 (naïve), and CD4-CCR6 (Th17) cells (**Figures 1C, D**).

Among these cell types, we observed a significantly increased proportion of cycling, mono-CD14^+^, mono-CD14^+^CD16^+^, CD4-FOXP3, and CD4-ICOS cells and a decreased fraction of mono-CD16^+^ cells, NK cells, T-CD8^+^ cells, γδT & MAIT cells, pDCs, and NKT cells in patients with severe disease compared with HCs (**Figures 1E, F**). Moreover, compared to patients with mild disease, patients with severe disease had higher proportions of mono-CD14^+^, mono-CD14^+^CD16^+^, and CD4-ICOS cells and lower proportions of NK cells, T-CD8^+^ cells, γδT & MAIT cells, pDCs, NKT cells, and CD8-CCR7 cells. These results are consistent with previous reports (19, 21), indicating that SARS-CoV-2 infection greatly perturbs the immune response.

To characterize the metabolic features of these disproportionate cell types in patients with COVID-19, we identified the DEGs (MAST algorithm; *P* < 0.01, logFC > 0.25 or logFC < -0.25) in patients with mild or severe disease compared to HCs and performed gene enrichment analysis (**Table S4**). The GO terms (*p*. adjust < 0.01) enriched with these DEGs overlapped with the child terms of metabolic processes (GO:0008152) (**Table S2** and **Figure 1G**). Monocytes and memory B cells showed significant metabolic changes, especially in patients with severe COVID-19. Cytokine production and ATP biosynthesis processes were dysfunctional in mono-CD14^+^ cells. Oxidative phosphorylation (OXPHOS) and protein processing were dysregulated in memory B cells. These findings demonstrate that metabolic changes in immune cells might play important roles in the control of the immune response in COVID-19 patients.

### Metabolic Transcriptome Rewiring in Immune Cells in COVID-19 Patients

**Figure 2:**
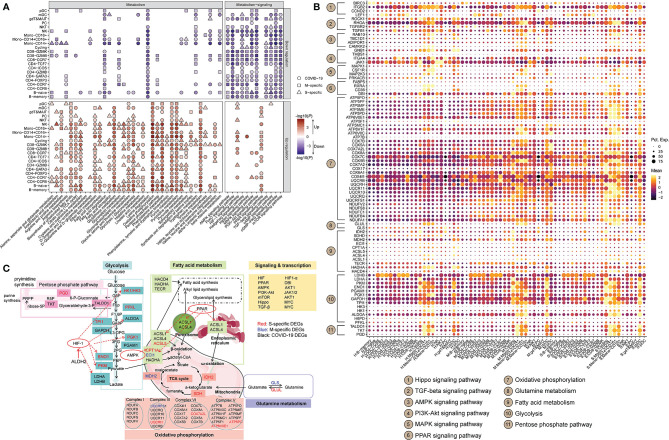
Metabolic transcriptome rewiring of immune cells in COVID-19 patients. **(A)** The significantly altered metabolic processes (left panel) and metabolism-related signaling (right panel) KEGG pathways in each cell cluster in COVID-19 patients (Student’s t-test; *P* < 0.01). M-specific: the unique KEGG pathways that were significantly altered in patients with mild disease compared to healthy controls. S-specific: the unique KEGG pathways that were significantly altered in patients with severe disease compared to healthy controls. COVID-19: KEGG pathways that were significantly altered in patients with mild and severe disease compared to healthy controls. **(B)** The dot plot of selected DEGs that are involved in metabolic processes or metabolism-related signaling KEGG pathways. “Mean” indicates the average gene expression level. “Pct.Exp.” indicates the percentage of cells expressing the corresponding genes. **(C)** Global maps of metabolic rewiring in COVID-19 patients according to the DEGs and the KEGG pathways in which they are involved. The S-specific DEGs are shown in red, the M-specific DEGs in blue, and the DEGs in COVID-19 patients in black.

A total of 36 metabolic pathways and 12 metabolism-related signaling pathways were analyzed (**Table S3**). For each cell, each pathway was scored according to the expression levels of its genes using Seurat (v3.2.2) (22). For each scored pathway, we performed Student’s t-test between HCs and mild or severe COVID-19 patient. Only terms with *P* < 0.01 are displayed. The DEGs in these metabolic pathways are listed in **Table S5**. Most of the metabolic pathways were more active in patients with COVID-19. However, metabolism-related signaling pathways were mostly downregulated in COVID-19 patients (**Figure 2A**). Compared to HCs, COVID-19 patients showed dramatic remodeling of several metabolic processes, including glucose, lipid metabolism, amino acid metabolism, nucleic acid metabolism, the tricarboxylic acid cycle (TCA cycle), and OXPHOS (**Figure 2A**). Of note, the majority of immune cells (~86.4%) in COVID-19 patients exhibited uniform and significant upregulation of the glycolytic process (**Figure 2A**). Specifically, in COVID-19 patients, mono-CD14^+^, and mono-CD14^+^CD16^+^ cells showed higher levels of glycolysis, fatty acid synthesis, TCA cycle activity, OXPHOS, and pentose phosphate pathway (PPP) activity with PPAR and HIF-1 signaling pathway activation than those in HCs, and these cells showed the highest PPP activity in patients with severe cases (**Figure 2A**). Moreover, after SARS-CoV-2 infection, alanine, aspartate and glutamate metabolism and lysine degradation were downregulated in mono-CD14^+^ cells. The genes involved in lysine degradation and in the AMPK, FoxO, AMPK, PI3K-AKT, mTOR, cAMP, and cGMP-PKG signaling pathways were downregulated in mono-CD14^+^CD16^+^ cells (**Figure 2A**). Mono-CD16^+^ cells exhibited higher glycolysis, fatty acid synthesis, TCA cycle, OXPHOS, PPP, and alanine, aspartate, glutamate and arginine metabolism activity in COVID-19 patients than in HCs; in addition, these cells exhibited lower lysine degradation activity and lower AMPK, Hippo, FoxO, AMPK, PI3K-AKT, mTOR, TGF-β, cAMP, and cGMP-PKG signaling pathway activity in COVID-19 patients than in HCs. In addition, mono-CD16^+^ cells from patients with severe COVID-19 showed particularly increased levels of alanine, aspartate and glutamate metabolism and fatty acid synthesis (**Figure 2A**).

Naïve and memory B cells in COVID-19 patients also displayed enhanced activity of glycolysis, fatty acid biosynthesis/elongation, the TCA cycle, OXPHOS, PPP, and metabolism of many amino acids, with PPAR pathway activation, than those in HCs. However, metabolism of alanine, aspartate, glutamate, arginine and lysine was downregulated in patients with COVID-19 (**Figure 2A**). Furthermore, the genes involved in glycolysis, fatty acid elongation, the TCA cycle, OXPHOS and arginine and proline metabolism were expressed at higher levels in PCs from COVID-19 patients than in PCs from HCs. These cells also showed activation of the PPAR pathway and inhibition of the MAPK and cGMP-PKG pathways. Severely ill COVID-19 patients had much higher glycolysis, fatty acid elongation, TCA cycle, arginine and proline metabolism activity in PCs than did HCs (**Figure 2A**).

The subsets of CD4^+^ T cells, *e.g.*, CD4-TCF7, CD4-ICOS, CD4-GATA3, CD4-CCR7 and CD4-CCR6 cells, showed similar alterations in metabolic processes, including enhanced glycolysis, TCA cycle activity, OXPHOS, arginine, proline, cysteine, methionine, glycine, serine, threonine and tyrosine metabolism; and reduced lysine degradation (**Figure 2A**). Additionally, CD8-GZMB cells mainly exhibited enhanced TCA cycle activity, OXPHOS, glycolysis, fatty acid degradation, and cysteine, methionine, glycine, serine, threonine, histidine and tyrosine metabolism in COVID-19 patients (**Figure 2A**). Metabolic changes in other subsets of CD4^+^ and CD8^+^ T cells were also observed in COVID-19 patients (**Figure 2A**). The signaling pathways regulating metabolic processes, including the TGF-beta signaling pathway, HIF-1 signaling pathway, FoxO signaling pathway, cAMP signaling pathway, and PI3K-Akt signaling pathway, were mostly downregulated in the subsets of CD4^+^ and CD8^+^ T cells in COVID-19 patients (**Figure 2A**).

After analyzing the genes participating in the metabolic processes and signaling pathways, we found that the immune cells exhibited cell type-specific transcriptomic signatures at the metabolic level (**Figures 2B, C**). Compared to HCs, patients with mild COVID-19 showed downregulation of a PPP-related gene (*TKT*), while patients in the severe COVID-19 group showed upregulation of glycolysis-related genes (*LDHA*, *PGD*, *PGAM1*, and *PKM*) and a fatty acid-related gene (*HACD4*) and downregulation of lysine degradation-related genes (*NSD1, SETD2, KMT2C, KMT2E* and *KMT2A*) in mono-CD14^+^ cells (**Figure 2B**). The mono-CD14^+^CD16^+^ cells in patients with mild COVID-19 expressed higher levels of a fatty acid-related gene (*FABP5*), whereas in patients with severe COVID-19, these cells expressed higher levels of glycolysis-related genes (*PKM, TALDO1*, and *LDHA*), a PPP-related genes (*PGD*), a fatty acid-related gene (*ASCL1*), a cysteine and methionine metabolism-related gene (*MAT2A*), and an alanine, aspartate and glutamate metabolic related gene (*GLUL*), as wells as lower levels of lysine metabolism-related genes (*KMT2C, SETD2, KMT2E* and *NSD1*). The CD16^+^ monocytes in the mild COVID-19 group expressed higher levels of glycolysis-related genes (*PGAM1* and *GAPDH*), a fatty acid-related gene (*HACD4*), and an arginine and proline metabolism-related gene (*SAT2*), as well as lower levels of a cysteine and methionine metabolism-related gene (*AHCYL1*) (**Figure 2B**). However, we found that CD16^+^ monocytes in patients with severe COVID-19 expressed higher levels of a PPP-related gene (*PGD*) and an arginine, alanine, aspartate and glutamate metabolism-related gene (*GLUL*), as well as lower levels of glycolysis-related genes (*HK1, PFKL, PGK1*, and *ENO1*), a fatty acid-related gene (*CPT1A*) and lysine metabolism-related genes (*ASH1L* and *NSD1*), an arginine and proline metabolism-related gene (*CKB*), and a tyrosine and phenylalanine metabolism-related gene (*COMT*) (**Figure 2B**). Genes involved in OXPHOS had marked transcriptional changes in all three subtypes of monocytes (**Figure 2B**). In CD14^+^ monocytes, HIF-1 signaling may regulate *LDHA*, *ALDOA*, *TIMP1*, *ELOB* and *IFNGR2*, and PPAR signaling may regulate *UBC*, *RXRA*, *DBI* and *ACSL1* in COVID-19 patients (**Figure 2C**). The detailed connections between metabolic processes and signaling pathways are shown in **Figure 2B, C**.

Based on these findings, a global metabolic reprogramming map of the peripheral immune cells of COVID-19 patients was constructed (**Figure 2C**). As shown, in COVID-19 patients, glycolysis-related enzymes, including *HK1/HK3*, *ALDOA*, *TPI1*, *PGK1*, *GAPDH, ENO1, PKM2*, and *LDHA/LAHB*, were significantly upregulated by HIF-1 signaling; the fatty acid metabolic genes *ASCL1, ASCL4* and *ASCL5* were upregulated by PPAR signaling; and CPT1A was upregulated by AMPK signaling. Thus, the metabolic transcriptome reprogramming in COVID-19 patients may be associated with immune system dysfunction.

### Dysfunction of Monocytes Was Connected With Metabolic Reprogramming in COVID-19 Patients

**Figure 3:**
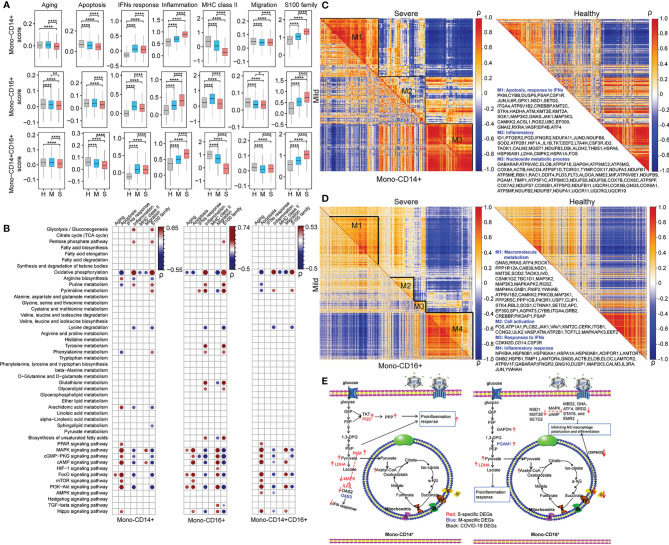
Dysfunction of monocytes was connected with metabolic reprogramming in COVID-19 patients. **(A)** The selected functional changes in mono-CD14^+^, mono-CD16^+^, and mono-CD14^+^CD16^+^ cells in patients with mild or severe disease (Student’s t-test; *P < 0.05; **P < 0.01; ****P < 0.0001). H, healthy controls; M, patients with mild COVID-19, and S, patients with severe COVID-19. **(B)** The Pearson correlations between the scores of monocyte function and metabolic processes and signaling pathways in mono-CD14^+^ (left panel), mono-CD16^+^ (middle panel) and mono-CD14^+^CD16^+^ (right panel) cells. “ρ” indicates the Pearson correlation coefficient; only dots representing correlations with **|**ρ**|** > 0.2 and *P* < 0.05 are shown. **(C, D)** The coexpression modules of DEGs in mono-CD14^+^ and mono-CD14^+^CD16^+^ cells. The metabolism-related genes are displayed. The coexpression modules in patients with mild and severe disease are shown in the lower triangle and upper triangle, respectively. The coexpression modules in healthy controls are shown in the right panel. “ρ” indicates the Pearson correlation coefficient. **(E)** The delineation of metabolism-immune response crosstalk in mono-CD14^+^ and mono-CD16^+^ cells in COVID-19 patients according to the DEGs and the KEGG pathways in which they participate. The S-specific DEGs are shown in red. The M-specific DEGs are shown in blue. The DEGs in COVID-19 patients are shown in black.

In this study, seven key biological processes involved in monocyte dysfunction in COVID-19 patients (4, 19, 21) were evaluated: aging, apoptosis, IFN response, inflammation, MHC class II, migration, and S100 family. Each process was scored using Seurat in each cell type in HCs, patients with mild COVID-19 and patients with severe COVID-19. After performing pairwise comparison using Student’s t-test, we found that inflammation, IFN response, and S100 family were significantly upregulated and apoptosis, migration, and MHC class II were significantly downregulated in both mono-CD14^+^ and mono-CD14^+^CD16^+^ cells in COVID-19 patients compared to the corresponding cells in HCs (**Figure 3A**). The IFN response in severe COVID-19 depends on sampling time (24, 25). In our cohort, severe COVID-19 with median 11.5 days from symptom onset showed impaired IFN response compared to that in mild COVID-19. Moreover, in these two cell types, patients with severe COVID-19 showed enhanced inflammation, migration, and S100 family and suppressed apoptosis and MHC class II compared with patients with mild COVID-19 (**Figure 3A**). Compared to patients with mild COVID-19, severely ill patients showed downregulation of IFN response in mono-CD14^+^ cells but upregulation of IFN response in mono-CD14^+^CD16^+^ cells (**Figure 3A**). These functional changes were consistent with previous reports (21).

To identify potential immune-metabolic interactions, we evaluated the Pearson correlation between the immune function score and metabolism score. Only correlations with *P <*0.05 and ρ>0.2 or ρ<-0.2 are displayed (**Table S6** and **Figure 3B**). Arginine biosynthesis, arachidonic acid metabolism, and the FoxO, Hippo, MAPK, PI3K-Akt, cAMP, cGMP-PKG, mTOR and PPAR pathways were positively correlated with apoptosis in mono-CD14+ cells, while phenylalanine, OXPHOS and purine metabolism were negatively correlated with apoptosis in mono-CD14^+^ cells. Moreover, glycolysis, PPP, OXPHOS and purine metabolism were positively associated with the inflammatory response and S100 family, whereas arginine biosynthesis, lysine degradation, arachidonic acid metabolism, and the MAPK, FoxO, PI3K-Akt, cAMP and cGMP-PGK pathways were negatively associated with the inflammatory response and S100 family in mono-CD14+ cells (**Figure 3B**). We found that tyrosine metabolism, glutathione metabolism, glycerolipid metabolism, biosynthesis of unsaturated fatty acids and pyrimidine metabolism were specifically positively correlated with inflammation and the S100 family in mono-CD16^+^. In addition, the correlations between lysine degradation and both inflammation and the S100 family were much stronger in mono-CD16^+^ cells than in mono-CD14^+^ cells. The correlations between metabolic processes and immune functions in mono-CD14^+^CD16^+^ cells are delineated in **Figure 3B**.

Strong correlations between the DEGs were observed in COVID-19 patients, especially in patients with severe disease, and weak correlations were observed in HCs (**Figure 3C**), suggesting that these genes may be activated after SARS-CoV-2 infection and that they tend to show a coordinated expression pattern or participate in common or similar biological processes (26). Specifically, three coexpression modules in COVID-19 patients were identified in mono-CD14^+^ cells (**Figure 3C** and **Table S7**). In module 1, *PKM*, with the highest expression in mono-CD14^+^ cells in patients with severe COVID-19, was found to be involved in glycolytic metabolism, which may suppress the IFN response by mediating IFN-related genes, such as *ILF3* (27), *OAS2* (28), and *OAS3* (29) (**Figures 3C, E**). In addition, *PKM* may participate in IFN response inhibition by suppressing MAPK pathway activation for its antiviral activity (30). In module 2, mono-CD14^+^ cells in patients with severe COVID-19 exhibited higher expression levels of glycolysis-related genes (*LDHA* and *PKM*) and PPP-related genes (*PGD* and *TKT*), which may be involved in the proinflammatory response (upregulation of *S100A8, S100A9, S100A12*, and *IL1B*) (**Figures 3C, E**). Enhanced glycolysis *via* upregulation of *LDHA* and *PKM* can promote inflammation *via* the HIF-1 signaling pathway (31) and can *also* fuel the PPP (*PGD* and *TKT*), which is vital for supporting the increased burden of protein, RNA and DNA synthesis in inflamed macrophages (32) (**Figures 3C, E**). The DEGs in module 3 were mainly involved in OXPHOS. These genes were correlated in similar patterns in HCs, patients with mild COVID-19 and patients with severe COVID-19 (**Figure 3C**).

For mono-CD16^+^ cells, we mainly analyzed modules 1 and 4 (**Figure 3D** and **Table S7**). In module 1, *NSD1, KMT2E*, and *SETD2*, which are histone lysine methyltransferases involved in cell development and differentiation (33), were expressed at lower levels in COVID-19 patients. The genes that participate in lysine degradation may inhibit the expression of genes related to M2 macrophage polarization and differentiation, such as *MBD2* (34), *GNAS* (35), *ATF4* (36), *BRD2* (37), *STAT6* (38), and *EMR2* (39), *via* the cAMP and MAPK signaling pathways (**Figures 3D, E**). In addition, module 1 contained genes involved in OXPHOS (*ATP6V1B2*, *ATP5A1*, *ATP5E*, and *ATP5B*), which may lead to M2-like polarization *via* the PI3K, AKT and mTOR signaling pathways (40, 41) (**Figures 3D, E**). These genes were significantly downregulated in patients with severe COVID-19, implying a limited ability for M2-like polarization. In module 4, enhanced glycolysis (increased *GAPDH* and *PGAM1* expression) in patients with mild COVID-19 mediated proinflammatory processes (such as S100 and TNF family) (42, 43) and the IFN-mediated antiviral response (44) in macrophages (**Figures 3D, E**). Enhanced OXPHOS was also found in mono-CD16^+^ cells from patients with severe COVID-19, broadening its physiological role as an antiviral agent (45).

### Metabolic Rewiring Correlate With the Differentiation, Immunoglobin Secretion and Survival of PCs and for the Differentiation of Memory B Cells in COVID-19 Patients

**Figure 4:**
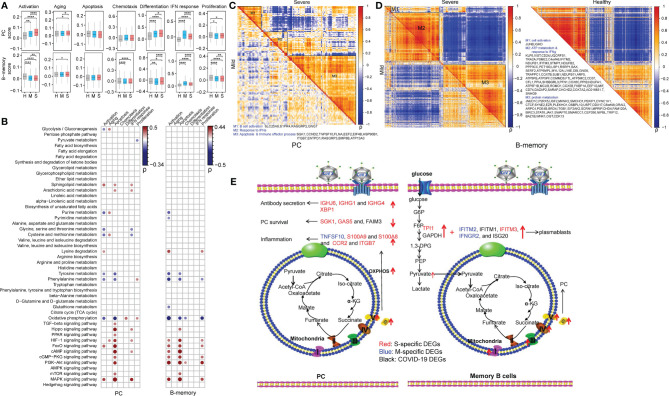
Metabolic rewiring correlate with the differentiation, immunoglobin secretion and survival of PCs and for the differentiation of memory B cells in COVID-19 patients. **(A)** The selected functional changes in PCs and memory B cells in COVID-19 patients (Student’s t-test; ^*^P < 0.05; ^**^P < 0.01; ^****^P < 0.0001); H, healthy control; M, patients with mild COVID-19; S, patients with severe COVID-19. **(B)** The Pearson correlations between the scores of B cell function and metabolic processes and signaling pathways in PCs (left panel) and memory B cells (right panel) of COVID-19 patients. “ρ” indicates the Pearson correlation coefficient; only dots representing correlations with |ρ| > 0.15 and *P* < 0.05 are shown. **(C, D)** Coexpression modules of DEGs in PCs and memory B cells in COVID-19 patients. The metabolism-related genes are marked. The coexpression modules in patients with mild and severe disease are shown in the lower triangle and upper triangle, respectively. “ρ” indicates the Pearson correlation coefficient. **(E)** The delineation of metabolism-immune response crosstalk in PCs and memory B cells in COVID-19 patients according to the DEGs and the KEGG pathways in which they participated. The S-specific DEGs are shown in red. The M-specific DEGs are shown in blue. The DEGs in COVID-19 patients are shown in black.

The regulatory roles of metabolic reprogramming in B cell function in COVID-19 patients were assessed, and functional alterations (aging, apoptosis, activation, differentiation, the IFN response, chemotaxis and proliferation) in PCs and memory B cells according to gene expression levels are summarized in **Figure 4A**. Also, pairwise comparisons using Student’s t-test revealed that genes involved in aging, activation, differentiation, and the IFN response were transcribed at higher levels in PCs of patients with mild and severe COVID-19 than in those of HCs, while genes related to proliferation were dramatically downregulated in COVID-19 patients (**Figure 4A**). Moreover, genes related to aging, activation, and differentiation were transcribed at higher levels in patients with severe COVID-19 than in patients with mild COVID-19, whereas genes involved in the IFN response were transcribed at lower levels (**Figure 4A**). The memory B cells of COVID-19 patients had enhanced aging and IFN response but reduced activation, chemotaxis, and proliferation compared to those of HCs (**Figure 4A**). Among patients with COVID-19, the severely ill group showed lower levels of genes related to activation, chemotaxis, the IFN response and proliferation (**Figure 4A**) than the mildly ill group.

We also calculated the correlation coefficients between these immune functions and the 48 metabolic processes in PCs and memory B cells (**Figure 4B** and **Table S8**). Here, we only show correlations with a *P* < 0.05 and ρ > 0.15 or ρ < -0.15. Lysine degradation and the FoxO and MAPK signaling pathways were positively correlated with the activation of memory B cells and PCs, and purine metabolism, tyrosine metabolism, phenylalanine metabolism and OXPHOS were negatively correlated with the activation of these two cell types. Tyrosine metabolism, phenylalanine metabolism and OXPHOS showed a negative correlation with apoptosis in PCs and memory B cells. Metabolic signaling pathways, *e.g.*, the Hippo, HIF-1, FoxO, cAMP, cGMP-PKG, PI3K-Akt, mTOR and MAPK signaling pathways, were found to be positively correlated with apoptosis in PCs and memory B cells. Of note, OXPHOS was positively correlated with cell differentiation specifically in PCs.

Gene coexpression analysis revealed that module 1 and module 2 had few metabolic changes in PCs in COVID-19 patients (**Table S9** and **Figure 4C**). Hence, we only analyzed module 3. In module 3, the OXPHOS-related gene (*ATP13A3*) was found to be positively correlated with genes encoding immunoglobin chains (*IGHJ6, IGHG1*, and *IGHG4*) and X-Box Binding Protein 1 (*XBP1*), which is required for plasmacytic differentiation and crucial for the plasma cell secretory program in patients with severe COVID-19 (**Figures 4C, E**) (46). The significant elevation of *XBP1* in patients with severe COVID-19 is important for the provision of additional extracellular amino acids, mitochondrial anaplerosis and cataplerosis, and subsequent sustained antibody secretion (47, 48). Enhanced OXPHOS was also linked to the expression of genes related to PC survival (apoptosis inhibition) (*SGK1* (49), *GAS5* (50), and *FAIM3* (51) (**Figure 4C**). Additionally, we found that PCs in patients with severe COVID-19 had higher expression levels of inflammation genes (*TNFSF10*, *S100A9*, and *S100A8*) and chemokine-related genes (*CCR2* and *ITGB7*) than those in patients with mild COVID-19. In memory B cells, module 1 and module 3 had few metabolic changes in COVID-19 patients, so we mainly analyzed module 2 (**Table S9** and **Figure 4D**). In module 2, enhanced expression of glycolysis (*GAPDH* and *TPI1*) and IFN response (*IFITM2*, *IFITM1*, *IFITM3*, *IFNGR2*, and *ISG20*) genes in human COVID-19 patients, particularly severely ill patients, may promote the differentiation of unswitched memory B cells into plasmablasts (**Figures 4D, E**) (52). In addition, elevated levels of OXPHOS-related genes, especially in patients with severe disease, may facilitate the differentiation of memory B cells into plasma cells (**Figures 4D, E**) (53).

### Metabolic Changes in T Cells in COVID-19 Patients Showed Common Characteristics

We scored the T cell functions, including the activation, aging, apoptosis, differentiation, IFN response and proliferation of T cells, in each T cell type. CD4-FOXP3 and CD4-ICOS cells showed downregulated apoptosis and differentiation and an upregulated IFN response in COVID-19 patients compared to HCs (**Figure S2A**). CD8-CCR7 and CD8-GZMB cells showed upregulated apoptosis and downregulated activation, differentiation, and proliferation in severely ill patients compared to HCs (**Figure S2A**). Consistent with the observations in monocytes, we found that patients with mild COVID-19 had a higher IFN response in these T cells than did patients with severe COVID-19 (**Figure S2A**). A correlation between metabolic processes and T cell immune functions was commonly observed in these T cell subtypes (**Table S10** and **Figure S2B**). The correlations with a *P* < 0.05 and ρ > 0.2 or ρ < -0.2 are highlighted. In brief, OXPHOS, tyrosine metabolism and phenylalanine metabolism were negatively correlated with T cell activation, apoptosis and differentiation. The MAPK, cAMP, FoxO and PI3K−Akt metabolic signaling pathways, were positively correlated with these functions. These data suggest that metabolic processes of T cells may be uniformly altered in COVID-19 patients. Hence, it is difficult to identify the metabolic rewiring responsible for the functional changes in a specific T cell type using single-cell RNA sequencing.

## Discussion

A hyperinflammatory status and impaired IFN response in mono-CD14^+^ cells and a decreased proportion and pathological inflammatory response of mono-CD16^+^ cells have been associated with the pathogenesis of severe COVID-19 (5, 54). However, the underlying metabolism-related mechanisms of these responses remain unclear. This study presents evidence of immunometabolic rewiring occurring in immune cells of COVID-19 patients. The transcriptional changes in genes in 36 metabolic processes and 12 metabolism-related signaling pathways were comprehensively explored and scored. Metabolic processes were mostly upregulated, with some upregulated metabolism-related signaling pathways, in COVID-19 patients.

The massive increase in mono-CD14^+^ cells in patients with severe COVID-19 could incite cytokine storm along with an impaired IFN response (6). However, the interferon response of severe COVID-19 depends on sampling time (24, 25). Severe COVID-19 tend to exhibit impaired IFN response in the early infection stage. In this study, we found that severe COVID-19 with median 11.5 days from symptom onset showed impaired IFN response compared to that in mild COVID-19. We analyzed the metabolic changes that were correlated with the impaired IFN response in severe COVID-19 in our cohort. Our results showed that the increased expression of *PKM* in monocytes of severely ill patients may suppress the expression of IFN-related genes, such as *ILF3*, *OAS2* and *OAS3*, by suppressing MAPK pathway activation (30). Moreover, we found enhanced glycolysis and PPP activity in mono-CD14^+^ cells of patients with severe COVID-19, which may be responsible for these inflammatory responses (55, 56). Additionally, mono-CD14^+^ cells in mildly ill COVID-19 patients showed enhanced glycolysis, which promoted their proinflammatory functions (42, 43) and IFN-mediated antiviral response. These findings suggest that metabolic remodeling contributes to hyperinflammation and impaired IFN response phenotypes in monocytes of patients with severe COVID-19. Future studies on association between metabolic changes and IFN response in other disease progression stages or in dynamic queues in COVID-19 patients are needed. Unlike mono-CD14^+^ cells, mono-CD16^+^ cells exhibit distinct motility within the vasculature and are considered patrolling monocytes. Both our study and previous studies observed that the fraction of mono-CD16^+^ cells was decreased in patients with severe COVID-19 compared with patients with mild disease or healthy controls (57). In mono-CD16^+^ cells in COVID-19 patients, we found reduced lysine degradation (downregulation of *NSD1*, *KMT2E*, and *SETD2)* and enhanced OXPHOS, which may inhibit M2 macrophage polarization and differentiation through the cAMP and MAPK signaling pathways or the PI3K, AKT and mTOR signaling pathways (40, 41). The observed enhancement of OXPHOS in patients with severe COVID-19 may broaden the pathological role of mono-CD16^+^ cells. Overall, these data indicate that metabolic changes play critical roles in the dysfunction of monocytes in COVID-19 patients. Further studies focusing on these crucial metabolism-related molecules, *e.g.*, *PKM, LDHA, PGAM1, PGD, TKT, NSD1, KMT2E, SETD2* and *OXPHOS-*related genes, are required to determine the benefits of ameliorating the monocyte-induced inflammatory response and inflammatory injury during SARS-CoV-2 infection.

The B cells of COVID-19 patients also showed enhanced OXPHOS and glycolysis. B cells from patients with severe disease showed obvious clonal expansion compared with those from patients with mild disease or healthy controls, indicating that B cell activity and humoral immune responses are strongly activated in patients with severe disease (58). Upon antigen recognition, B cells become activated, which requires increased glucose uptake (59, 60). Naïve B cells depend on OXPHOS for their survival (61), indicating that these metabolic adaptations can support the proliferation and activation of naïve B cells. The observed enhancement of OXPHOS in PCs could promote the processes of differentiation, antibody secretion, and cell survival in patients with severe disease (46, 62). It is worth noting that we found that enhanced OXPHOS was positively correlated with the expression of *XBP1*, whose transcription in PCs of patients with severe disease could significantly increase mitochondrial mass and mitochondrial respiration (63) and is essential for maintaining energy homeostasis and durable humoral immunity (47). In addition, increased respiratory capacity characterized by enhanced OXPHOS is also required for the long-term survival of PCs (47). Additionally, we found that PCs produced many inflammatory factors. Because RBD-specific IgG1 and IgG3 dominate the humoral response against SARS-CoV-2 infection and are positively associated with inflammation (64), future studies should address the roles of metabolic adaptations in PCs in the promotion of IgG1 production and the exacerbation of the disease. In memory B cells, we identified several potential genes related to OXPHOS that may facilitate the differentiation of memory B cells into plasma cells in COVID-19 patients, especially those with severe disease (52). Future studies should focus on OXPHOS-associated antibody production and proinflammatory processes in PCs of patients with severe COVID-19.

The subsets of CD4^+^ and CD8^+^ T cells in COVID-19 patients also exhibited an increased demand for OXPHOS and amino acid metabolism, indicating their involvement in T cell maturation and proliferation (65) However, we did not find unique metabolic characteristics in any of the T cell subtypes, implying the limited power of this approach to analyze metabolism in T cells of COVID-19 patients. More technologies or strategies are needed to analyze immunometabolic rewiring in T cells after SARS-COV-2 infection.

Taken together, these data provide a landscape of the dysfunctional metabolic reprogramming in peripheral immune cells, revealing the potential metabolic mechanisms responsible for the immune response in COVID-19. However, metabolite-based experimental technologies are needed to verify our findings in future studies.

## Conclusions

The metabolic landscape of peripheral immune cells during SARS-CoV-2 infection was comprehensively mapped and assessed. We found that enhanced glycolysis and PPP activity are potential metabolic mechanisms underlying the impaired IFN response and hyperinflammation in mono-CD14^+^ cells. In addition, the inhibited lysine degradation and enhanced OXPHOS in mono-CD16^+^ cells might inhibit M2 macrophage polarization and differentiation. The enhanced OXPHOS and glycolysis in plasma cells were also explored and found to play critical roles in antibody production and survival of PCs. These metabolic adaptations in immune cells are important in the immune hyperactivation and immunopathogenesis of COVID-19.

## Data Availability Statement

Publicly available datasets were analyzed in this study. This data can be found here: The single-cell gene expression data of PBMCs from 21 COVID-19 patients (10 with mild cases and 11 with severe cases) and 11 healthy controls (HCs) were downloaded from the GEO database (https://www.ncbi.nlm.nih.gov/geo/) or GSA database (https://bigd.big.ac.cn/gsa/). The corresponding accession numbers were GSE150728 (19), GSE149689 (20) and HRA000297 (21).

## Ethics Statement

Ethical review and approval was not required for the study on human participants in accordance with the local legislation and institutional requirements. Written informed consent for participation was not required for this study in accordance with the national legislation and the institutional requirements.

## Author Contributions

FQ and WZ collected the data and performed data analysis, JH and LF revised the figures and tables. JZ and FQ drew the figures and wrote the manuscript. All authors contributed to the article and approved the submitted version.

## Funding

The study was supported by the Central Charity Fund of Chinese Academy of Medical Science (2020-PT310-009) and the China Postdoctoral Science Foundation (no. 2020T130122ZX to FQ, no. 2020M670289 and no. 2020T130065ZX to JZ).

## Conflict of Interest

The authors declare that the research was conducted in the absence of any commercial or financial relationships that could be construed as a potential conflict of interest

## Supplementary Material

Supplementary Figure 1 The coexpression gene modules in mono-CD14^+^CD16^+^ cells. The metabolism-related genes are displayed. The coexpression modules in patients with mild and severe disease are shown in the lower triangle and upper triangle, respectively. The coexpression modules in healthy controls are shown in the right panel. “ρ” indicates the Pearson correlation coefficient.

Supplementary Figure 2 Metabolic changes in T cells in COVID-19 patients showed common characteristics. **(A)** The selected functional changes in CD4-ICOS, CD4-FOXP3, CD8-CCR7, and CD8-GZMB cells in COVID-19 patients (Student’s t-test; **P* < 0.05; ***P* < 0.01; ****P* < 0.001); H: healthy controls, M: patients with mild COVID-19, S: patients with severe COVID-19. **(B)** The Pearson correlations between the scores of T cell function and metabolic processes and signaling pathways in CD4-ICOS, CD4-FOXP3, CD8-CCR7, and CD8-GZMB cells. “ρ” indicates the Pearson correlation coefficient. Only dots representing correlations with **|**ρ**|** > 0.2 and *P* < 0.05 are shown.
