# Supplementary figures and images for: Single-Cell RNA Sequencing Analysis of the Immunometabolic Rewiring and Immunopathogenesis of Coronavirus Disease 2019
Source: Front Immunol. 2021 Apr 14;12:651656. doi: 10.3389/fimmu.2021.651656 (PMC8079812; doi:10.3389/fimmu.2021.651656)

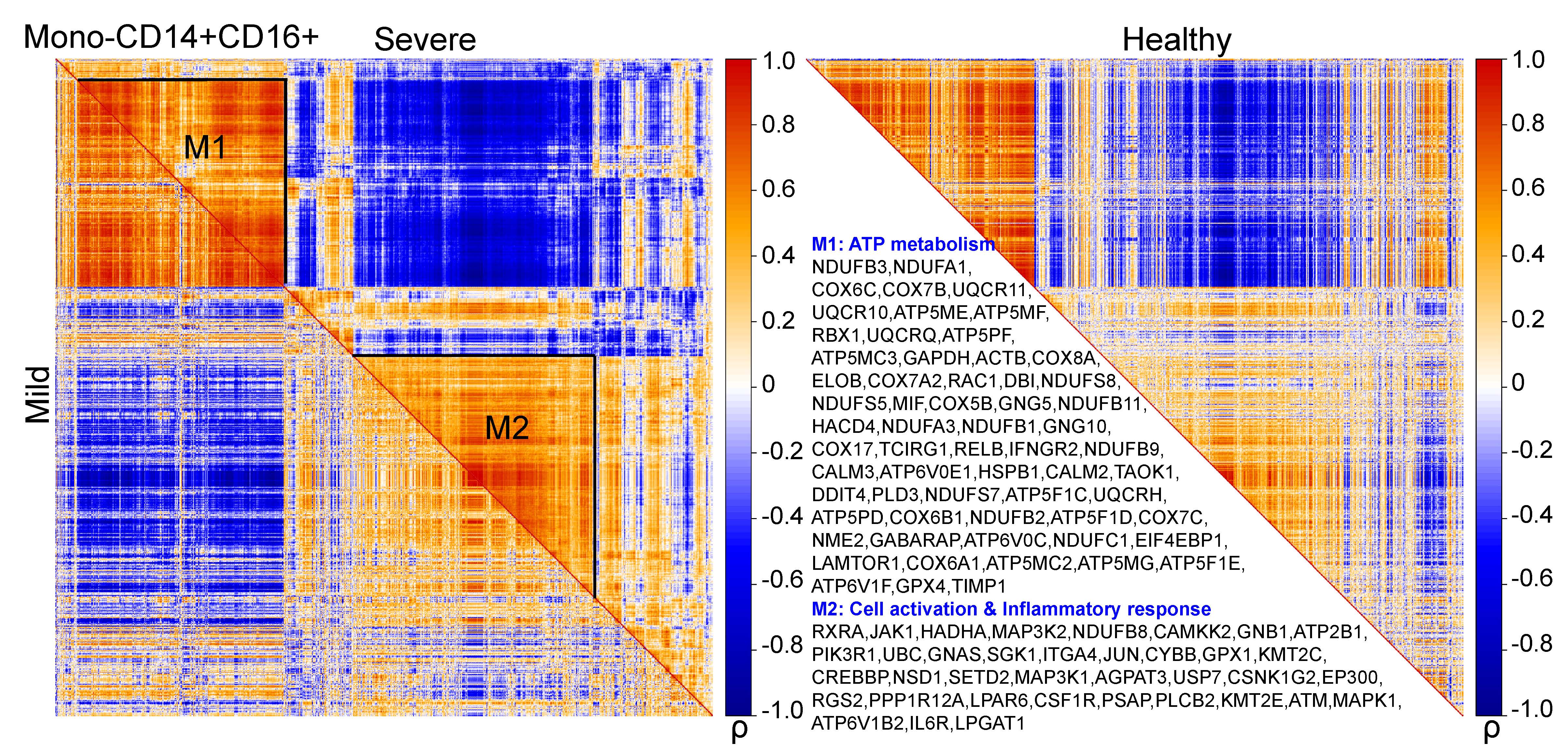

Supplement: Supplementary Figure 1 — The coexpression gene modules in mono-CD14+CD16+ cells. The metabolism-related genes are displayed. The coexpression modules in patients with mild and severe disease are shown in the lower triangle and upper triangle, respectively. The coexpression modules in healthy controls are shown in the right panel. “ρ” indicates the Pearson correlation coefficient. [file Image_1.jpeg]

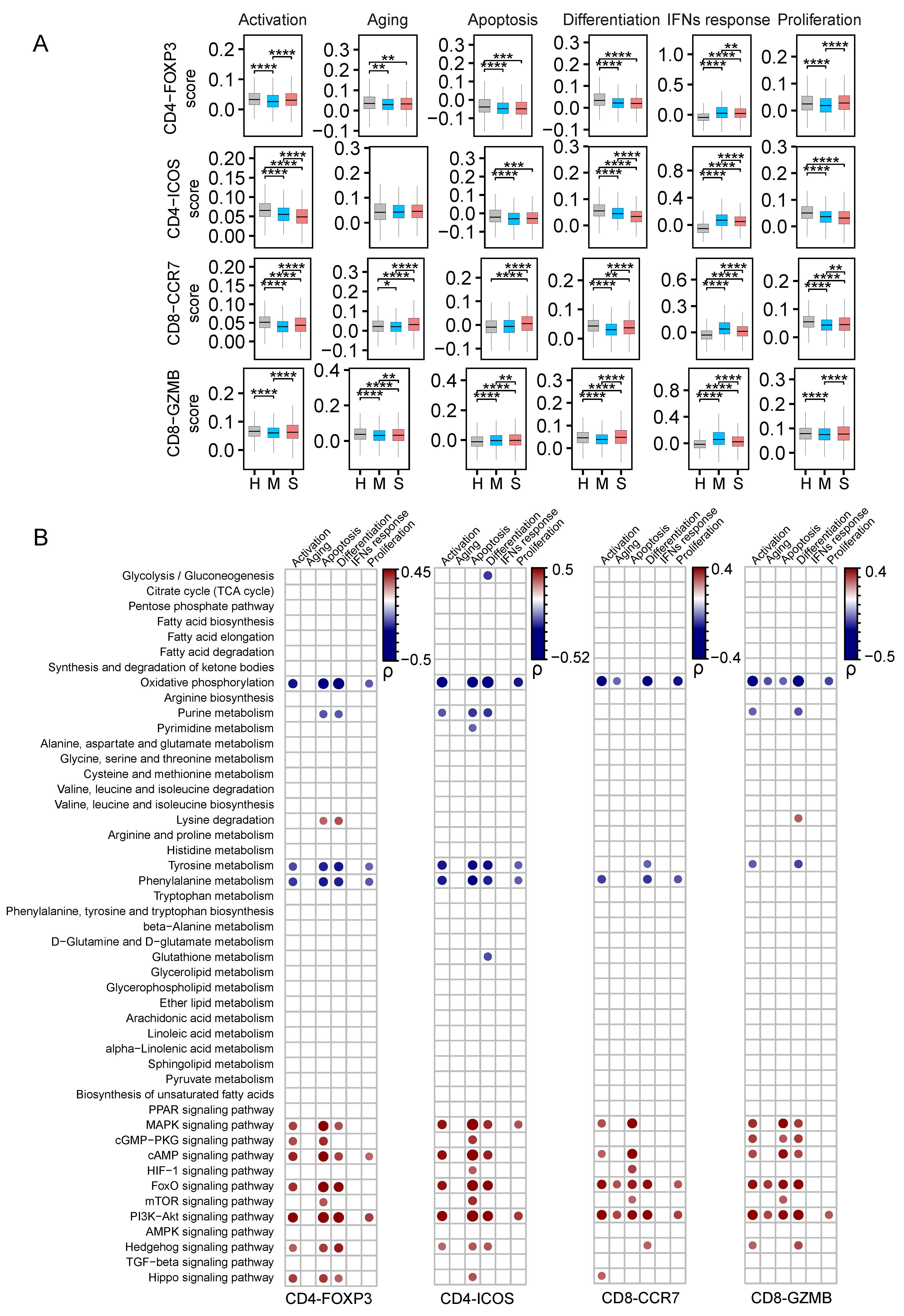

Supplement: Supplementary Figure 2 — Metabolic changes in T cells in COVID-19 patients showed common characteristics. (A) The selected functional changes in CD4-ICOS, CD4-FOXP3, CD8-CCR7, and CD8-GZMB cells in COVID-19 patients (Student’s t-test; *P < 0.05; **P < 0.01; ***P < 0.001); H: healthy controls, M: patients with mild COVID-19, S: patients with severe COVID-19. (B) The Pearson correlations between the scores of T cell function and metabolic processes and signaling pathways in CD4-ICOS, CD4-FOXP3, CD8-CCR7, and CD8-GZMB cells. “ρ” indicates the Pearson correlation coefficient. Only dots representing correlations with |ρ| > 0.2 and P < 0.05 are shown. [file Image_2.jpeg]
