# Supplementary material for: Single-Cell RNA Sequencing Analysis of the Immunometabolic Rewiring and Immunopathogenesis of Coronavirus Disease 2019
Source: Front Immunol. 2021 Apr 14;12:651656. doi: 10.3389/fimmu.2021.651656 (PMC8079812; doi:10.3389/fimmu.2021.651656)
Supplement: Supplementary file 3 [file DataSheet_1.docx]

**Data integration**

library(Seurat)

library(Matrix)

library(dplyr)

library(ggplot2)

library(MAST)

ob.list=list()

sample1=c('C7','C8','M2','M3','M4','M5','M6','S5','S6','S7','S8','S9','S10','S11')

for(i in 1:length(sample1)){

sampleID <- sample1[i]

cellranger_pipestance_path <- paste("./Data/",sampleID,sep="")

data <- Read10X(cellranger_pipestance_path)

data.seurat <- CreateSeuratObject(counts = data, project = sampleID, min.cells = 10,min.features = 200)

data.seurat[['percent.mito']] <- PercentageFeatureSet(data.seurat, pattern = "^MT-")

data.seurat$sample <- sampleID

data.filter <- subset(x = data.seurat, subset = nFeature_RNA > 200 & nFeature_RNA < 6000 & nCount_RNA > 1000 & nCount_RNA < 15000 & percent.mito < 15)

data.filter <- NormalizeData(object = data.filter, verbose = FALSE)

data.filter <- FindVariableFeatures(object = data.filter, selection.method = "vst", nfeatures = 2000)

ob.list = append(ob.list,data.filter)

}

sample2=c('C1','C2','C3','C4','C5','C6','M1','M9','M10','S1','S2','S3')

for(i in 1:length(sample2)){

subm=readRDS(paste('./Data/',sample2[i],'/',sample2[i],'.rds',sep=''))

data.seurat <- CreateSeuratObject(counts = as.matrix(subm$exon), project = sample2[i], min.cells = 10,min.features = 200)

data.seurat[['percent.mito']] <- PercentageFeatureSet(data.seurat, pattern = "^MT-")

data.seurat$sample <- sample2[i]

data.filter <- subset(x = data.seurat, subset = nFeature_RNA > 200 & nFeature_RNA < 6000 & nCount_RNA > 1000 & nCount_RNA < 15000 & percent.mito < 15)

data.filter <- NormalizeData(object = data.filter, verbose = FALSE)

data.filter <- FindVariableFeatures(object = data.filter, selection.method = "vst", nfeatures = 2000)

ob.list = append(ob.list,data.filter)

}

sample3=c('C9','C10','C11','M7','M8','S4')

for(i in 1:length(sample3)){

exp=read.table(paste('./Data/',sample3[i],'/','rawcount_matrix.txt',sep=''))

data.seurat <- CreateSeuratObject(counts = as.matrix(exp), project = sample3[i], min.cells = 10,min.features = 200)

data.seurat[['percent.mito']] <- PercentageFeatureSet(data.seurat, pattern = "^MT-")

data.seurat$sample <- sample3[i]

data.filter <- subset(x = data.seurat, subset = nFeature_RNA > 200 & nFeature_RNA < 6000 & nCount_RNA > 1000 & nCount_RNA < 15000 & percent.mito < 15)

data.filter <- NormalizeData(object = data.filter, verbose = FALSE)

data.filter <- FindVariableFeatures(object = data.filter, selection.method = "vst", nfeatures = 2000)

ob.list = append(ob.list,data.filter)

}

anchors <- FindIntegrationAnchors(object.list = ob.list, dims = 1:50,reference=3)

combined <- IntegrateData(anchorset = anchors, dims = 1:50)

saveRDS(combined,file='combined.rds')

scale.combined <- ScaleData(object = combined, vars.to.regress = c("nCount_RNA", "percent.mito"))

pca.combined <- RunPCA(object = scale.combined, npcs = 50,verbose = FALSE,features=VariableFeatures(scale.combined))

umap.combined <- FindNeighbors(object = pca.combined, reduction = "pca",dims=1:20)

umap.combined <- FindClusters(umap.combined, resolution = 1)

umap.combined <- RunUMAP(object = umap.combined, reduction = "pca", dims = 1:20)

umap.combined@meta.data$group[umap.combined@meta.data$sample == "C1"] = "H"

umap.combined@meta.data$group[umap.combined@meta.data$sample == "C2"] = "H"

umap.combined@meta.data$group[umap.combined@meta.data$sample == "C3"] = "H"

umap.combined@meta.data$group[umap.combined@meta.data$sample == "C4"] = "H"

umap.combined@meta.data$group[umap.combined@meta.data$sample == "C5"] = "H"

umap.combined@meta.data$group[umap.combined@meta.data$sample == "C6"] = "H"

umap.combined@meta.data$group[umap.combined@meta.data$sample == "C7"] = "H"

umap.combined@meta.data$group[umap.combined@meta.data$sample == "C8"] = "H"

umap.combined@meta.data$group[umap.combined@meta.data$sample == "C9"] = "H"

umap.combined@meta.data$group[umap.combined@meta.data$sample == "C10"] = "H"

umap.combined@meta.data$group[umap.combined@meta.data$sample == "C11"] = "H"

umap.combined@meta.data$sample[umap.combined@meta.data$sample == "C1"] = "H1"

umap.combined@meta.data$sample[umap.combined@meta.data$sample == "C2"] = "H2"

umap.combined@meta.data$sample[umap.combined@meta.data$sample == "C3"] = "H3"

umap.combined@meta.data$sample[umap.combined@meta.data$sample == "C4"] = "H4"

umap.combined@meta.data$sample[umap.combined@meta.data$sample == "C5"] = "H5"

umap.combined@meta.data$sample[umap.combined@meta.data$sample == "C6"] = "H6"

umap.combined@meta.data$sample[umap.combined@meta.data$sample == "C7"] = "H7"

umap.combined@meta.data$sample[umap.combined@meta.data$sample == "C8"] = "H8"

umap.combined@meta.data$sample[umap.combined@meta.data$sample == "C9"] = "H9"

umap.combined@meta.data$sample[umap.combined@meta.data$sample == "C10"] = "H10"

umap.combined@meta.data$sample[umap.combined@meta.data$sample == "C11"] = "H11"

umap.combined@meta.data$group[umap.combined@meta.data$sample == "M1"] = "M"

umap.combined@meta.data$group[umap.combined@meta.data$sample == "M2"] = "M"

umap.combined@meta.data$group[umap.combined@meta.data$sample == "M3"] = "M"

umap.combined@meta.data$group[umap.combined@meta.data$sample == "M4"] = "M"

umap.combined@meta.data$group[umap.combined@meta.data$sample == "M5"] = "M"

umap.combined@meta.data$group[umap.combined@meta.data$sample == "M6"] = "M"

umap.combined@meta.data$group[umap.combined@meta.data$sample == "M7"] = "M"

umap.combined@meta.data$group[umap.combined@meta.data$sample == "M8"] = "M"

umap.combined@meta.data$group[umap.combined@meta.data$sample == "M9"] = "M"

umap.combined@meta.data$group[umap.combined@meta.data$sample == "M10"] = "M"

umap.combined@meta.data$group[umap.combined@meta.data$sample == "S1"] = "S"

umap.combined@meta.data$group[umap.combined@meta.data$sample == "S2"] = "S"

umap.combined@meta.data$group[umap.combined@meta.data$sample == "S3"] = "S"

umap.combined@meta.data$group[umap.combined@meta.data$sample == "S4"] = "S"

umap.combined@meta.data$group[umap.combined@meta.data$sample == "S5"] = "S"

umap.combined@meta.data$group[umap.combined@meta.data$sample == "S6"] = "S"

umap.combined@meta.data$group[umap.combined@meta.data$sample == "S7"] = "S"

umap.combined@meta.data$group[umap.combined@meta.data$sample == "S8"] = "S"

umap.combined@meta.data$group[umap.combined@meta.data$sample == "S9"] = "S"

umap.combined@meta.data$group[umap.combined@meta.data$sample == "S10"] = "S"

umap.combined@meta.data$group[umap.combined@meta.data$sample == "S11"] = "S"

saveRDS(umap.combined,file='all.rds')

png(file="umap.png", width=900,height=800)

DimPlot(object = umap.combined, reduction = 'umap',label = TRUE,label.size=8)+NoLegend()

dev.off()

markers <- FindAllMarkers(object = umap.combined, only.pos = TRUE,test.use='MAST',assay='RNA',slot='data')

write.table(markers,file='All.markers.txt',row.names = FALSE,quote = FALSE,sep = '\t')

**Figure plot**

library(Seurat)

library(MAST)

library(ggplot2)

library(tidyverse)

library(concaveman)

library(ggforce)

library(deldir)

library(clusterProfiler)

library(org.Hs.eg.db)

library(enrichplot)

allcolor=c("#FF1493","#0000CD","#008B8B","#FFE4B5","#8A2BE2","#228B22","#E9967A","#4682B4","#32CD32","#F0E68C","#EE82EE","#FF6347","#6A5ACD","#9932CC","#8B008B","#8B4513","#DEB887","#20B2AA","#FFA500","#9370DB","#98FB98","#F08080","#1E90FF","#7CFC00","#FFFF00","#808000","#FF00FF","#FA8072","#7B68EE","#9400D3","#800080","#A0522D","#D2B48C","#D2691E","#87CEEB","#40E0D0","#5F9EA0")

##Figure 1A

subc=readRDS('all.rds')

pdf('all-umap.pdf',width=5,height=5)

subc<-AddMetaData(subc,subc@reductions$umap@cell.embeddings,col.name = colnames(subc@reductions$umap@cell.embeddings))

class_avg <-subc@meta.data %>% group_by(CellType) %>% summarise(UMAP_1 = median(UMAP_1),UMAP_2 = median(UMAP_2))

p=ggplot(subc@meta.data ,aes(x=UMAP_1,y=UMAP_2))+geom_point(aes(color=CellType),size=0.02)+scale_color_manual(values = allcolor)+geom_text(aes(label = CellType),size=6,data = class_avg)

p=p+theme_bw()+theme(panel.border = element_blank(),panel.grid=element_blank(),axis.title = element_blank(),axis.text = element_blank(),axis.line = element_blank(),axis.ticks = element_blank(),legend.position = 'none')

print(p)

dev.off()

##Figure 1B

subm=readRDS('all.rds')

pdf('all-marker.pdf',width=7,height=4)

subm$CellType=factor(subm$CellType,levels = c('HSC','NK','T-CD8+','T-CD4+','Mono-CD14+','Mono-CD16+','Mono-CD14+CD16+','mDC','pDC','B-naive','B-memory','PC','gdT&MAIT','Cycling'))

gene=c('CD34','KLRF1','CD3D','CD8A','CD4','CD14','FCGR3A','CD1C','LILRA4','CD79A','MS4A1','TCL1A','IGKC','TRGV9','SLC4A10','MKI67')

DefaultAssay(subm)='RNA'

DotPlot(subm,features=gene,group.by='CellType',cols=c('grey','#DC143C'))+theme(axis.title = element_blank(),axis.text.x = element_text(angle=90,vjust=0.5,hjust=1))+scale_color_viridis_c(option='B')

dev.off()

##Figure 1C

subc=readRDS('tcell.rds')

pdf('tcell-umap.pdf',width=7,height=7)

subc<-AddMetaData(subc,subc@reductions$umap@cell.embeddings,col.name = colnames(subc@reductions$umap@cell.embeddings))

class_avg <-subc@meta.data %>% group_by(TcellType) %>% summarise(UMAP_1 = median(UMAP_1),UMAP_2 = median(UMAP_2))

p=ggplot(subc@meta.data ,aes(x=UMAP_1,y=UMAP_2))+geom_point(aes(color=TcellType),size=0.01,pch=20)+scale_color_manual(values = allcolor,name='')+geom_text(aes(label = TcellType),size=6,data = class_avg)

p=p+theme_bw()+theme(panel.border = element_blank(),panel.grid=element_blank(),axis.title = element_blank(),axis.text = element_blank(),axis.line = element_blank(),axis.ticks = element_blank(),legend.position = 'none')

print(p)

dev.off()

##Figure 1D

subm=readRDS('tcell.rds')

subm$TcellType=factor(subm$TcellType,levels = c("CD4-CCR6","CD4-CCR7","CD4-FOXP3","CD4-GATA3","CD4-GZMB","CD4-ICOS","CD4-TCF7","CD8-CCR7","CD8-GZMB","CD8-GZMK","NKT"))

DefaultAssay(subm)='RNA'

genes=c('CD4','CCR6','CCR7','FOXP3','GATA3','GZMB','ICOS','TCF7','CD8A','GZMK','KLRF1')

pdf('tcell-marker.pdf',width=5.5,height=3.5)

DotPlot(subm,features=genes,group.by='TcellType')+theme(axis.title = element_blank(),axis.text.x = element_text(angle=90,vjust=0.5,hjust=1))+scale_color_viridis_c(option='B')

dev.off()

##Figure 1E

library(reshape2)

subc=readRDS('all.rds')

num=as.matrix(table(subc$CellType,subc$sample))

prop=apply(num,2,function(x)x*100/sum(x))

a=melt(prop)

colnames(a)=c('CellType','Sample','Prop')

a$Group=substr(a$Sample,1,1)

my_comparisons=list(c('M','H'),c('S','H'),c('S','M'))

pdf('all.bar.pdf',width=8,height=4)

p=ggbarplot(a, x="Group", y="Prop",fill='Group',add = c("mean_se"),facet.by = "CellType",ncol=7,add.params = list(size=0.1),position = position_dodge(0.8),size=0.1, legend='none',scales='free_y' ,xlab='',ylab='Cell fraction (%)',title='')+rotate_x_text(angle = 0)

p=p+stat_compare_means(comparisons=my_comparisons,method = "t.test",label = "p.signif",hide.ns = TRUE,vjust=0.5)

p=p+scale_fill_manual(values=c('grey','DeepSkyBlue','LightCoral'),name='')

print(p)

dev.off()

##Figure 1F

library(reshape2)

subc=readRDS('tcell.rds')

num=as.matrix(table(subc$TcellType,subc$sample))

prop=apply(num,2,function(x)x*100/sum(x))

a=melt(prop)

colnames(a)=c('CellType','Sample','Prop')

a$Group=substr(a$Sample,1,1)

my_comparisons=list(c('M','H'),c('S','H'),c('S','M'))

pdf('tcell.bar.pdf',width=8,height=4)

p=ggbarplot(a, x="Group", y="Prop",fill='Group',add = c("mean_se"),facet.by = "CellType",ncol=7,add.params = list(size=0.1),position = position_dodge(0.8),size=0.1, legend='none',scales='free_y' ,xlab='',ylab='Cell fraction (%)',title='')+rotate_x_text(angle = 0)

p=p+stat_compare_means(comparisons=my_comparisons,method = "t.test",label = "p.signif",hide.ns = TRUE,vjust=0.5)

p=p+scale_fill_manual(values=c('grey','DeepSkyBlue','LightCoral'),name='')

print(p)

dev.off()

##Figure 1G

subc=readRDS('all.rds')

num<-table(pbmc@meta.data$subType,pbmc@meta.data$group)

types=unique(pbmc$subType)

cd14=matrix(nrow=0,ncol=12)

for (i in c(1:length(types))){

subcell=subset(pbmc,subType==types[i])

if(num[types[i],'M']>3 && num[types[i],'H']>3){

a=gsub('/','-',types[i])

tmp1=FindMarkers(subcell, ident.1='M', ident.2 = 'H', group.by = 'group',test.use='MAST',assay='RNA',slot='data',logfc.threshold = 0,min.pct = 0.2)

write.table(tmp1,file=paste("M.to.H.",a,".DEGs.txt",sep=""),quote=FALSE,sep="\t",row.names=TRUE,col.names=TRUE)

#tmp1=read.table(file=paste("COVID-19.to.HC.",a,".DEGs.txt",sep=""),sep='\t',head=T,row.names=1)

tmp11=subset(tmp1,avg_logFC>0.25 & p_val_adj<0.01)

genes=as.character(unique(rownames(tmp11)))

genes=genes[!grepl("^MT-|^RP[SL]", genes)]

IDs = mget(genes,revmap(org.Hs.egSYMBOL),ifnotfound=NA)

BP <- enrichGO(gene = IDs,OrgDb=org.Hs.eg.db,ont = "BP",pAdjustMethod = "BH",minGSSize = 1,pvalueCutoff = 0.01,qvalueCutoff = 0.05,readable = TRUE)

#BP <- enrichKEGG(gene=IDs,keyType="kegg",organism='hsa',pvalueCutoff=0.01,pAdjustMethod="BH",qvalueCutoff=0.05)

bp11=BP@result

bp11$Type='up'

bp11$CellType=types[i]

bp11$Compare='M.H'

tmp12=subset(tmp1,avg_logFC<(-0.25) & p_val_adj<0.01)

genes=as.character(unique(rownames(tmp12)))

genes=genes[!grepl("^MT-|^RP[SL]", genes)]

IDs = mget(genes,revmap(org.Hs.egSYMBOL),ifnotfound=NA)

BP <- enrichGO(gene = IDs,OrgDb=org.Hs.eg.db,ont = "BP",pAdjustMethod = "BH",minGSSize = 1,pvalueCutoff = 0.01,qvalueCutoff = 0.05,readable = TRUE)

#BP <- enrichKEGG(gene=IDs,keyType="kegg",organism='hsa',pvalueCutoff=0.01,pAdjustMethod="BH",qvalueCutoff=0.05)

bp12=BP@result

bp12$Type='down'

bp12$CellType=types[i]

bp12$Compare='M.H'

deg=rbind(bp11,bp12)

cd14=rbind(cd14,deg)

}

if(num[types[i],'S']>3 && num[types[i],'H']>3){

a=gsub('/','-',types[i])

tmp1=FindMarkers(subcell, ident.1='S', ident.2 = 'H', group.by = 'group',test.use='MAST',assay='RNA',slot='data',logfc.threshold = 0,min.pct = 0.2)

write.table(tmp1,file=paste("S.to.H.",a,".DEGs.txt",sep=""),quote=FALSE,sep="\t",row.names=TRUE,col.names=TRUE)

#tmp1=read.table(file=paste("COVID-19.to.HC.",a,".DEGs.txt",sep=""),sep='\t',head=T,row.names=1)

tmp11=subset(tmp1,avg_logFC>0.25 & p_val_adj<0.01)

genes=as.character(unique(rownames(tmp11)))

genes=genes[!grepl("^MT-|^RP[SL]", genes)]

IDs = mget(genes,revmap(org.Hs.egSYMBOL),ifnotfound=NA)

BP <- enrichGO(gene = IDs,OrgDb=org.Hs.eg.db,ont = "BP",pAdjustMethod = "BH",minGSSize = 1,pvalueCutoff = 0.01,qvalueCutoff = 0.05,readable = TRUE)

#BP <- enrichKEGG(gene=IDs,keyType="kegg",organism='hsa',pvalueCutoff=0.01,pAdjustMethod="BH",qvalueCutoff=0.05)

bp11=BP@result

bp11$Type='up'

bp11$CellType=types[i]

bp11$Compare='S.H'

tmp12=subset(tmp1,avg_logFC<(-0.25) & p_val_adj<0.01)

genes=as.character(unique(rownames(tmp12)))

genes=genes[!grepl("^MT-|^RP[SL]", genes)]

IDs = mget(genes,revmap(org.Hs.egSYMBOL),ifnotfound=NA)

BP <- enrichGO(gene = IDs,OrgDb=org.Hs.eg.db,ont = "BP",pAdjustMethod = "BH",minGSSize = 1,pvalueCutoff = 0.01,qvalueCutoff = 0.05,readable = TRUE)

#BP <- enrichKEGG(gene=IDs,keyType="kegg",organism='hsa',pvalueCutoff=0.01,pAdjustMethod="BH",qvalueCutoff=0.05)

bp12=BP@result

bp12$Type='down'

bp12$CellType=types[i]

bp12$Compare='S.H'

deg=rbind(bp11,bp12)

cd14=rbind(cd14,deg)

}

if(num[types[i],'S']>3 && num[types[i],'M']>3){

a=gsub('/','-',types[i])

tmp1=FindMarkers(subcell, ident.1='S', ident.2 = 'M', group.by = 'group',test.use='MAST',assay='RNA',slot='data',logfc.threshold = 0,min.pct = 0.2)

write.table(tmp1,file=paste("S.to.M.",a,".DEGs.txt",sep=""),quote=FALSE,sep="\t",row.names=TRUE,col.names=TRUE)

#tmp1=read.table(file=paste("COVID-19.to.HC.",a,".DEGs.txt",sep=""),sep='\t',head=T,row.names=1)

tmp11=subset(tmp1,avg_logFC>0.25 & p_val_adj<0.01)

genes=as.character(unique(rownames(tmp11)))

genes=genes[!grepl("^MT-|^RP[SL]", genes)]

IDs = mget(genes,revmap(org.Hs.egSYMBOL),ifnotfound=NA)

BP <- enrichGO(gene = IDs,OrgDb=org.Hs.eg.db,ont = "BP",pAdjustMethod = "BH",minGSSize = 1,pvalueCutoff = 0.01,qvalueCutoff = 0.05,readable = TRUE)

bp11=BP@result

bp11$Type='up'

bp11$CellType=types[i]

bp11$Compare='S.M'

tmp12=subset(tmp1,avg_logFC<(-0.25) & p_val_adj<0.01)

genes=as.character(unique(rownames(tmp12)))

genes=genes[!grepl("^MT-|^RP[SL]", genes)]

IDs = mget(genes,revmap(org.Hs.egSYMBOL),ifnotfound=NA)

BP <- enrichGO(gene = IDs,OrgDb=org.Hs.eg.db,ont = "BP",pAdjustMethod = "BH",minGSSize = 1,pvalueCutoff = 0.01,qvalueCutoff = 0.05,readable = TRUE)

bp12=BP@result

bp12$Type='down'

bp12$CellType=types[i]

bp12$Compare='S.M'

deg=rbind(bp11,bp12)

cd14=rbind(cd14,deg)

}

}

write.table(cd14,file="all.BP.txt",quote=FALSE,sep="\t",row.names=FALSE,col.names=TRUE)

b=read.table('metabolic_Go.txt',sep='\t')

a=read.table('all.BP.txt',sep='\t',head=T,row.names=NULL,quote="")

met=as.vector(b$V1)

a$metbo=ifelse(a$ID %in% met,'Metabolism','Other')

a=subset(a,Count>4 & p.adjust<0.05)

e=subset(a,metbo!='Other')

e$group[e$Type == 'up']=1

e$group[e$Type == 'down']=-1

e$p.adjust=as.numeric(e$p.adjust)

write.table(e,file="all.BP.flt.txt",quote=FALSE,sep="\t",row.names=FALSE,col.names=TRUE)

e=read.table('all.BP.flt-1.txt',sep='\t',head=TRUE,row.names=NULL,quote="")

pdf('all.BP.pdf',width=17,height=9)

p=ggplot(e,aes(x=CellType,y=Description,fill=log(-log10(p.adjust)),shape=Fun))+geom_point(color='black',size=3)+theme_bw()+theme(panel.grid = element_blank())

p=p+facet_wrap(.~Type,ncol=1,strip.position='right')

p=p+scale_fill_viridis_c(option='B')+coord_flip()+scale_shape_manual(values=c(21,22,24))

p=p+theme(axis.title = element_blank(),axis.text.x = element_text(angle=60,color = 'black',vjust=1,hjust=1),axis.text.y = element_text(color = 'black'))

print(p)

dev.off()

##Figure 2A

a=read.table('metabolism.term.txt',sep='\t')

b=read.table('KEGG.txt',sep='\t')

colnames(a)=c('ID','Term','Category')

colnames(b)=c('ID','Term','GeneID','Gene')

term=unique(a$ID)

suba=readRDS('all.rds')

DefaultAssay(suba)='RNA'

for(i in 1:length(term)){

su=subset(b,ID==as.character(term[i]))

suba=AddModuleScore(object = suba,features = list(su$Gene),name=term[i],assay = 'RNA',ctrl = 10)

}

score=suba@meta.data

types=paste(term,'1',sep='')

cells=unique(score$subType)

ps=matrix(nrow=0,ncol=4)

for(i in c(1:length(cells))){

tmp=subset(score,subType==cells[i])

h=subset(tmp,group=='H')

m=subset(tmp,group=='M')

s=subset(tmp,group=='S')

for(j in 1:length(types)){

pv1=t.test(m[,types[j]],h[,types[j]])

pv2=t.test(s[,types[j]],h[,types[j]])

pvd1=data.frame(cells[i],types[j],pv1$p.value,(pv1$estimate[1]-pv1$estimate[2]))

pvd2=data.frame(cells[i],types[j],pv2$p.value,(pv2$estimate[1]-pv2$estimate[2]))

pvd1$Compare='M.H'

pvd2$Compare='S.H'

colnames(pvd1)=c('cells','types','pvalue','diff','Compare')

colnames(pvd2)=c('cells','types','pvalue','diff','Compare')

pvd=rbind(pvd1,pvd2)

ps=rbind(ps,pvd)

}

}

ps$group[ps$diff>0]='up'

ps$group[ps$diff<0]='down'

ps$group[ps$diff==0]='none'

write.table(ps,file='score.pvalue.all.txt',sep='\t',row.names = F,col.names = T,quote = F)

ps=read.table('score.pvalue.all.txt',sep='\t',head=T)

ps=subset(ps,pvalue<0.001)

ps$groups[ps$diff>0]=1

ps$groups[ps$diff<0]=-1

ps$groups[ps$diff==0]=0

ps$logp=log(-log10(ps$pvalue))

ps$logps=(ps$groups)*(ps$logp)

ps$ID=substr(ps$types,1,8)

psall=merge(ps,a,by='ID')

write.table(psall,file='score.pvalue.all.plus.term.txt',sep='\t',row.names = F,col.names = T,quote = F)

psall=read.table('score.pvalue.all.plus.term-1.txt',sep='\t',quote="",head=TRUE,row.names=NULL)

pdf('score.point.pdf',width=11,height=9)

p=ggplot(psall,aes(x=Term,y=cells,fill=logps,color=group,shape=Fun))+geom_point(stroke=0.3,size=3.5)+scale_fill_gradient2(low='navy',mid='white',high='#8B0000',midpoint=0)

p=p+theme_bw()+theme(panel.grid = element_blank(),axis.text = element_text(color='black'))+scale_shape_manual(values=c(21,22,24))+facet_grid(group~Category,scales='free_x',space='free_x')

p=p+theme(axis.text.x = element_text(angle=45,vjust=1,hjust=1))+labs(x='',y='')+scale_color_manual(values=c('black','black'))

print(p)

dev.off()

##Figure 2B

suba=readRDS('all.rds')

DefaultAssay(suba)='RNA'

suba@meta.data$IDS=paste(suba@meta.data$group,suba@meta.data$subType,sep=':')

cells=unique(suba$subType)

degall=matrix(nrow=0,ncol=8)

for(i in 1:length(cells)){

deg1=read.table(paste('M.to.H.',cells[i],'.DEGs.txt',sep=''),sep='\t',head=TRUE,row.names=1)

deg2=read.table(paste('S.to.H.',cells[i],'.DEGs.txt',sep=''),sep='\t',head=TRUE,row.names=1)

deg1$Gene=rownames(deg1)

deg2$Gene=rownames(deg2)

deg1s=subset(deg1,p_val_adj<0.01 & abs(avg_logFC)>0.25)

deg2s=subset(deg2,p_val_adj<0.01 & abs(avg_logFC)>0.25)

c=intersect(rownames(deg1s),rownames(deg2s))

m=setdiff(rownames(deg1s),c)

s=setdiff(rownames(deg2s),c)

deg=rbind(deg1s,deg2s)

deg$Compare=ifelse(deg$Gene %in% s,'S-specific',ifelse(deg$Gene %in% m,'M-specific','COVID-19'))

deg$CellType=cells[i]

degall=rbind(degall,deg)

}

write.table(degall,file='all.DEGs.txt',sep='\t',row.names = F,col.names = T,quote = F)

a=read.table('metabolism.term.txt',sep='\t')

b=read.table('KEGG.txt',sep='\t')

colnames(a)=c('ID','Term','Category')

colnames(b)=c('ID','Term','GeneID','Gene')

ab=merge(a,b,by=c('ID','Term'))

degab=subset(ab,Gene %in% degall$Gene)

write.table(degab,file='Metabolism.DEGs.txt',sep='\t',row.names = F,col.names = T,quote = F)

gene1=c('PGD','TKT','TALDO1','PFKL','H6PD','ALDOA','HK1','HK3','TPI1','GAPDH','PGK1','PGAM1','ENO1','PKM','LDHA','LDHB','HACD4','HADHA','TECR','ACSL1','ACSL4','ACSL5','CPT1A','ECI1','MDH2','SDHD','IDH2','GLS','GLUL','NDUFA1','NDUFB4','NDUFC1','NDUFS8','NDUFV2',

'UQCRFS1','UQCRQ','UQCR10','UQCR11','UQCRH','UQCRB','COX4I1','COX6A1','COX17','COX7A2','COX5B','COX7C','COX8A','COX7A2L','COX5A','COX7B','ATP7B','ATP6V0C','ATP6V1F','ATP5MC1','ATP5F1','ATP6V0E1','ATP5PD','ATP5ME','ATP5MF','ATP5PF','ATP5PO','DBI','CD36','RXRA','FABP5','PRKACB','MAP2K3','CSF1R','MAPK1','JAK1','ITGA4','THBS1','GNB1','CAMKK2','ADIPOR1','TBC1D1','RAB10','TGFB1','TGFBR2','RHOA','ROCK1','LEF1','CCND2','ITGB2','BIRC3')

pdf('degs.metabolic.pdf',width=15,height=16)

DotPlot(suba,features=gene1,group.by='IDS')+theme(axis.title = element_blank(),axis.text.x = element_text(angle=45,vjust=1,hjust=1))+scale_color_viridis_c(option='B')+coord_flip()

dev.off()

##Figure 3A

Inflammation = list(c('IFNG', 'IL10', 'IL12A', 'IL13', 'IL17A', 'IL18','IL1A', 'IL1B', 'IL2', 'IL21', 'IL22', 'IL23A', 'IL4', 'IL5', 'IL6', 'S100A8', 'S100A9','S100A10','S100A11','S100A6','S100A12','TNF','CXCL8'))

IFNresponse =list(c('ADAR','APOBEC3','BST2','CD74','MB21D1','DDIT4','DDX58','DDX60','EIF2AK2','GBP1','GBP2','HPSE','IFI44L','IFI6','IFIH1','IFIT1','IRF1','IRF7','ISG15','ISG20','MAP3K14','MOV10','MS4A4A','MX1','MX2','NAMPT','NT5C3','OAS1','OAS2','OAS3','OASL','P2RY6','PHF15','PML','RSAD2','RTP4','SLC15A3','SLC25A28','SSBP3','TREX1','TRIM5','TRIM25','SUN2','ZC3HAV1','IFITM1','IFITM2','IFITM3'))

MHC = list(c('HLA-DMA','HLA-DMB','HLA-DPA1','HLA-DPB1','HLA-DQA1','HLA-DQB1','HLA-DRA','HLA-DRB1','HLA-DRB5'))

S100= list(c('S100A1','S100A2','S100A3','S100A4','S100A5','S100A6','S100A7','S100A7A','S100A7L2','S100A7P1','S100A7P2','S100A8','S100A9','S100A10','S100A11','S100A12','S100A13','S100A14','S100A15A','S100A16','S100B','S100G','S100P','S100Z'))

Apoptosis= list(c('TNFSF10', 'TNFRSF10A','TNFRSF10B','FASLG','FAS','FADD','TNF','TNFRSF1A','TRADD','CFLAR','CASP8','CASP10','CASP6','CASP3','CASP7','BID','BAX','BAK1','DIABLO','SEPTIN4','HTRA2','CYCS','APAF1','CASP9','PRF1','GZMB','TUBA1B','TUBA4A','TUBA3C','TUBA1A','TUBA1C','TUBA8','TUBA3E','TUBA3D','TUBAL3','MCL1','ACTG1','ACTB','SPTA1','SPTAN1','LMNA','LMNB1','LMNB2','PARP1','PARP2','PARP3','PARP4','DFFA','DFFB','ENDOG','AIFM1','ERN1','TRAF2','ITPR1','ITPR2','ITPR3','CAPN1','CAPN2','CASP12','EIF2AK3','EIF2S1','ATF4','DDIT3','CTSB','CTSC','CTSD','CTSF','CTSH','CTSK','CTSL','CTSO','CTSS','CTSV','CTSW','CTSZ','BIRC2','BIRC3','XIAP','BIRC5','BCL2L11','BCL2L1','BCL2','DAXX','RIPK1','DAB2IP','MAP3K5','MAPK8','MAPK10','MAPK9','BAD','JUN','FOS','TP53','HRK','MAP3K14','CHUK','IKBKB','IKBKG','NFKBIA','NFKB1','RELA','PTPN13','GADD45A','GADD45B','GADD45G','TRAF1','BCL2A1','ATM','PIDD1','TP53AIP1','BBC3','PMAIP1','CASP2','NGF','NTRK1','IL3','IL3RA','CSF2RB','PIK3CA','PIK3CD','PIK3CB','PIK3R1','PIK3R2','PIK3R3','PDPK1','AKT1','AKT2','AKT3','HRAS','KRAS','NRAS','RAF1','MAP2K1','MAP2K2','MAPK1','MAPK3'))

migration =list(c('PECAM1','GAS6','IL4','PLCB1','APP','APOD','PTK2B','HMGB1','TMEM102','GATA3','CCL17','C1QBP','GCSAM','CD99L2','MSMP','ARHGEF5','JAM2','MSTN','TNFSF11','CXCR4','XG','ANXA1','CCL7','CCL8','DUSP1','ZAP70','S100A14','SLC8B1','MAPK1','C5AR1','CKLF','CCL4','PDGFB','PTK2','CCL3L1','CCL22','CMKLR1','CCN3','PIK3CG','TNFRSF14','AKT1','CCL19','KLRK1','MDK','DEFB104A','CXCL17','TNF','EPS8','XCL1','S1PR1','RARRES2','CCL21','SLC12A2','AIF1','FPR2','CXCR2','CXCR1','ITGA4','CCL18','CCL23','ADAM10','CALCA','CCL13','ANO6','CCR2','IL12A','IL34','WNK1','CD99','ARTN','MYO1G','PDGFD','FADD','AKIRIN1','CXCL10','PLA2G7','GREM1','PADI2','CDC42','THBS1','RET','LYN','ICAM1','FLT1','LGALS3','CSF1','CRKL','CRK','CD200R1','TRPM4','KARS1','NBL1','LGALS9','MADCAM1','CCL16','CCL25','RHOA','WASL','GPR15L','JAML','LGMN','IL27RA','CCL24','WNT5A','SPNS2','CD200','CX3CL1','MSN','ITGB7','CXCL11','ALOX5','PIK3CD','SLIT2','STK10','CCR6','CCR5','CCL11','S100A7','AGER','CXCL12','TRPM2','ADAM8','SIRPA','CCL20','ADAM17','SAA1','SERPINE1','TBX21','CCL14','ECM1','RIPOR2','HSD3B7','CCL1','CXCL16','F11R','CD47','PYCARD','IL6R','CCL5','CCL2','S100A12','GPR15','CXCR3','FUT7','CXCL14','CXCL13','AIRE','OXSR1','SPN','ITGAL','SELENOK','CRTAM','CREB3','CCL26','CSF1R','MAPK14','PLG','TNFRSF11A','RPS19','CH25H','DEFB124','CCL4L1','BMP5','SLAMF8','CCL15','XCL2','ADTRP','IL6','CYP7B1','MAPK3','CCL27','RIPK3','MOSPD2','MIA3','GPR183','CCR7','CCR1','STK39','GCSAML','DEFA1','CALR','NLRP12','C3AR1','LRCH1','CCL3','DOCK8','PTPRO'))

tmp=read.table('Cell-aging.txt')

Aging=list(tmp$V1)

subm=readRDS('all.rds')

DefaultAssay(subm)='RNA'

types=c(Inflammation,IFNresponse,MHC,S100,Apoptosis,migration,Aging)

names=c('Inflammation','IFNs response','MHC class II','S100 family','Apoptosis','Migration','Aging')

ps=matrix(nrow=0,ncol=6)

cells=c('Mono-CD14+','Mono-CD16+','Mono-CD14+CD16+')

for(i in 1:length(cells)){

subc=subset(subm,subType==cells[i])

for(j in 1:length(names)){

subc=AddModuleScore(object = subc, features = types[j],ctrl = 10,name = 'score')

tmp=subc@meta.data[,c(5,7,8,13,14)]

colnames(tmp)=c('sample','seurat_clusters','group','subType','score')

tmp$Type=names[j]

ps=rbind(ps,tmp)

}

}

write.table(ps,file='Monocyte-immune.score.txt',sep='\t',row.names = T,col.names = T,quote = F)

ps=read.table('Monocyte-immune.score.txt',sep='\t',head=T,row.names=1)

ps1=subset(ps,subType=='Mono-CD14+')

ps2=subset(ps,subType=='Mono-CD16+')

ps3=subset(ps,subType=='Mono-CD14+CD16+')

my_comparisons=list(c('M','H'),c('S','H'),c('S','M'))

pdf('monocyte-immune.score.pdf',width=8,height=8)

p1=ggboxplot(ps1, x="group", y="score",fill='group', size=0.1,facet.by = "Type",ncol=7,width=0.6, strip.position='top',legend = "none",scales='free_y',outlier.shape = NA,xlab='',ylab='Mono-CD14+')+rotate_x_text(angle = 0)

p1=p1+stat_compare_means(comparisons=my_comparisons,method = "t.test",label = "p.signif",hide.ns = TRUE,vjust=0.5)

p1=p1+scale_fill_manual(values=c('grey','DeepSkyBlue','LightCoral'),name='')

p2=ggboxplot(ps2, x="group", y="score",fill='group', size=0.1,facet.by = "Type",ncol=7,width=0.6, strip.position='top',legend = "none",scales='free_y',outlier.shape = NA,xlab='',ylab='Mono-CD16+')+rotate_x_text(angle = 0)

p2=p2+stat_compare_means(comparisons=my_comparisons,method = "t.test",label = "p.signif",hide.ns = TRUE,vjust=0.5)

p2=p2+scale_fill_manual(values=c('grey','DeepSkyBlue','LightCoral'),name='')

p3=ggboxplot(ps3, x="group", y="score",fill='group', size=0.1,facet.by = "Type",ncol=7,width=0.6, strip.position='top',legend = "none",scales='free_y',outlier.shape = NA,xlab='',ylab='Mono-CD14+CD16+')+rotate_x_text(angle = 0)

p3=p3+stat_compare_means(comparisons=my_comparisons,method = "t.test",label = "p.signif",hide.ns = TRUE,vjust=0.5)

p3=p3+scale_fill_manual(values=c('grey','DeepSkyBlue','LightCoral'),name='')

p=ggarrange(p1,p2,p3,ncol=1)

print(p)

dev.off()

##Figure S2A

Apoptosis= list(c('TNFSF10', 'TNFRSF10A','TNFRSF10B','FASLG','FAS','FADD','TNF','TNFRSF1A','TRADD','CFLAR','CASP8','CASP10','CASP6','CASP3','CASP7','BID','BAX','BAK1','DIABLO','SEPTIN4','HTRA2','CYCS','APAF1','CASP9','PRF1','GZMB','TUBA1B','TUBA4A','TUBA3C','TUBA1A','TUBA1C','TUBA8','TUBA3E','TUBA3D','TUBAL3','MCL1','ACTG1','ACTB','SPTA1','SPTAN1','LMNA','LMNB1','LMNB2','PARP1','PARP2','PARP3','PARP4','DFFA','DFFB','ENDOG','AIFM1','ERN1','TRAF2','ITPR1','ITPR2','ITPR3','CAPN1','CAPN2','CASP12','EIF2AK3','EIF2S1','ATF4','DDIT3','CTSB','CTSC','CTSD','CTSF','CTSH','CTSK','CTSL','CTSO','CTSS','CTSV','CTSW','CTSZ','BIRC2','BIRC3','XIAP','BIRC5','BCL2L11','BCL2L1','BCL2','DAXX','RIPK1','DAB2IP','MAP3K5','MAPK8','MAPK10','MAPK9','BAD','JUN','FOS','TP53','HRK','MAP3K14','CHUK','IKBKB','IKBKG','NFKBIA','NFKB1','RELA','PTPN13','GADD45A','GADD45B','GADD45G','TRAF1','BCL2A1','ATM','PIDD1','TP53AIP1','BBC3','PMAIP1','CASP2','NGF','NTRK1','IL3','IL3RA','CSF2RB','PIK3CA','PIK3CD','PIK3CB','PIK3R1','PIK3R2','PIK3R3','PDPK1','AKT1','AKT2','AKT3','HRAS','KRAS','NRAS','RAF1','MAP2K1','MAP2K2','MAPK1','MAPK3'))

Cytotoxicity=list(c('PRF1', 'IFNG', 'GNLY', 'NKG7', 'GZMB', 'GZMA', 'GZMH', 'KLRK1', 'KLRB1', 'KLRD1', 'CTSW', 'CST7'))

Exhaustion=list(c('LAG3', 'TIGIT', 'PDCD1', 'CTLA4', 'HAVCR2', 'TOX'))

migration =list(c('APP','APOD','TMEM102','CD99L2','XG','ZAP70','PIK3CG','TNFRSF14','XCL1','S1PR1','CCL21','SLC12A2','AIF1','ITGA4','ADAM10','CCR2','WNK1','CD99','MYO1G','FADD','CXCL10','ICAM1','CRKL','CRK','CD200R1','RHOA','IL27RA','WNT5A','CD200','MSN','ITGB7','CXCL11','PIK3CD','CCR6','S100A7','CXCL12','ADAM8','CCL20','ADAM17','RIPOR2','CXCL16','F11R','PYCARD','CCL5','CCL2','GPR15','CXCR3','CXCL13','AIRE','OXSR1','SPN','ITGAL','SELENOK','CCL26','XCL2','CCL27','RIPK3','GPR183','STK39','DEFA1','LRCH1','CCL3','DOCK8'))

IFNresponse =list(c('ADAR','APOBEC3','BST2','CD74','MB21D1','DDIT4','DDX58','DDX60','EIF2AK2','GBP1','GBP2','HPSE','IFI44L','IFI6','IFIH1','IFIT1','IRF1','IRF7','ISG15','ISG20','MAP3K14','MOV10','MS4A4A','MX1','MX2','NAMPT','NT5C3','OAS1','OAS2','OAS3','OASL','P2RY6','PHF15','PML','RSAD2','RTP4','SLC15A3','SLC25A28','SSBP3','TREX1','TRIM5','TRIM25','SUN2','ZC3HAV1','IFITM1','IFITM2','IFITM3'))

tmp=read.table('T-cell-activation.txt')

Act=list(tmp$V1)

tmp=read.table('T-cell-differentiation.txt')

Dif=list(tmp$V1)

tmp=read.table('T-cell-proliferation.txt')

Pro=list(tmp$V1)

tmp=read.table('Cell-aging.txt')

Aging=list(tmp$V1)

subm=readRDS('all.rds')

DefaultAssay(subm)='RNA'

types=c(Act,Aging,Apoptosis,Dif,IFNresponse,Pro)

names=c('Activation','Aging','Apoptosis','Differentiation','IFNs response','Proliferation')

ps=matrix(nrow=0,ncol=6)

cells=c('CD4-FOXP3','CD4-ICOS','CD8-CCR7','CD8-GZMB')

for(i in 1:length(cells)){

subc=subset(subm,subType==cells[i])

for(j in 1:length(names)){

subc=AddModuleScore(object = subc, features = types[j],ctrl = 10,name = 'score')

tmp=subc@meta.data[,c(5,7,8,13,14)]

colnames(tmp)=c('sample','seurat_clusters','group','subType','score')

tmp$Type=names[j]

ps=rbind(ps,tmp)

}

}

write.table(ps,file='T-immune.score.txt',sep='\t',row.names = T,col.names = T,quote = F)

ps=read.table('T-immune.score.txt',sep='\t',head=T,row.names=1)

ps1=subset(ps,subType=='CD4-FOXP3')

ps2=subset(ps,subType=='CD4-ICOS')

ps3=subset(ps,subType=='CD8-CCR7')

ps4=subset(ps,subType=='CD8-GZMB')

pdf('T-immune.score.pdf',width=6,height=7)

my_comparisons=list(c('M','H'),c('S','H'),c('S','M'))

p1=ggboxplot(ps1, x="group", y="score",fill='group', size=0.1,facet.by = "Type",ncol=7,width=0.6, strip.position='top',legend = "none",scales='free_y',outlier.shape = NA,xlab='',ylab='CD4-FOXP3')+rotate_x_text(angle = 0)

p1=p1+stat_compare_means(comparisons=my_comparisons,method = "t.test",label = "p.signif",hide.ns = TRUE,vjust=0.5)

p1=p1+scale_fill_manual(values=c('grey','DeepSkyBlue','LightCoral'),name='')

p2=ggboxplot(ps2, x="group", y="score",fill='group', size=0.1,facet.by = "Type",ncol=7,width=0.6, strip.position='top',legend = "none",scales='free_y',outlier.shape = NA,xlab='',ylab='CD4-ICOS')+rotate_x_text(angle = 0)

p2=p2+stat_compare_means(comparisons=my_comparisons,method = "t.test",label = "p.signif",hide.ns = TRUE,vjust=0.5)

p2=p2+scale_fill_manual(values=c('grey','DeepSkyBlue','LightCoral'),name='')

p3=ggboxplot(ps3, x="group", y="score",fill='group', size=0.1,facet.by = "Type",ncol=7,width=0.6, strip.position='top',legend = "none",scales='free_y',outlier.shape = NA,xlab='',ylab='CD8-CCR7')+rotate_x_text(angle = 0)

p3=p3+stat_compare_means(comparisons=my_comparisons,method = "t.test",label = "p.signif",hide.ns = TRUE,vjust=0.5)

p3=p3+scale_fill_manual(values=c('grey','DeepSkyBlue','LightCoral'),name='')

p4=ggboxplot(ps4, x="group", y="score",fill='group', size=0.1,facet.by = "Type",ncol=7,width=0.6, strip.position='top',legend = "none",scales='free_y',outlier.shape = NA,xlab='',ylab='CD8-GZMB')+rotate_x_text(angle = 0)

p4=p4+stat_compare_means(comparisons=my_comparisons,method = "t.test",label = "p.signif",hide.ns = TRUE,vjust=0.5)

p4=p4+scale_fill_manual(values=c('grey','DeepSkyBlue','LightCoral'),name='')

p=ggarrange(p1,p2,p3,p4,ncol=1)

print(p)

dev.off()

##Figure 4A

IFNresponse =list(c('ADAR','APOBEC3','BST2','CD74','MB21D1','DDIT4','DDX58','DDX60','EIF2AK2','GBP1','GBP2','HPSE','IFI44L','IFI6','IFIH1','IFIT1','IRF1','IRF7','ISG15','ISG20','MAP3K14','MOV10','MS4A4A','MX1','MX2','NAMPT','NT5C3','OAS1','OAS2','OAS3','OASL','P2RY6','PHF15','PML','RSAD2','RTP4','SLC15A3','SLC25A28','SSBP3','TREX1','TRIM5','TRIM25','SUN2','ZC3HAV1','IFITM1','IFITM2','IFITM3'))

Apoptosis= list(c('TNFSF10', 'TNFRSF10A','TNFRSF10B','FASLG','FAS','FADD','TNF','TNFRSF1A','TRADD','CFLAR','CASP8','CASP10','CASP6','CASP3','CASP7','BID','BAX','BAK1','DIABLO','SEPTIN4','HTRA2','CYCS','APAF1','CASP9','PRF1','GZMB','TUBA1B','TUBA4A','TUBA3C','TUBA1A','TUBA1C','TUBA8','TUBA3E','TUBA3D','TUBAL3','MCL1','ACTG1','ACTB','SPTA1','SPTAN1','LMNA','LMNB1','LMNB2','PARP1','PARP2','PARP3','PARP4','DFFA','DFFB','ENDOG','AIFM1','ERN1','TRAF2','ITPR1','ITPR2','ITPR3','CAPN1','CAPN2','CASP12','EIF2AK3','EIF2S1','ATF4','DDIT3','CTSB','CTSC','CTSD','CTSF','CTSH','CTSK','CTSL','CTSO','CTSS','CTSV','CTSW','CTSZ','BIRC2','BIRC3','XIAP','BIRC5','BCL2L11','BCL2L1','BCL2','DAXX','RIPK1','DAB2IP','MAP3K5','MAPK8','MAPK10','MAPK9','BAD','JUN','FOS','TP53','HRK','MAP3K14','CHUK','IKBKB','IKBKG','NFKBIA','NFKB1','RELA','PTPN13','GADD45A','GADD45B','GADD45G','TRAF1','BCL2A1','ATM','PIDD1','TP53AIP1','BBC3','PMAIP1','CASP2','NGF','NTRK1','IL3','IL3RA','CSF2RB','PIK3CA','PIK3CD','PIK3CB','PIK3R1','PIK3R2','PIK3R3','PDPK1','AKT1','AKT2','AKT3','HRAS','KRAS','NRAS','RAF1','MAP2K1','MAP2K2','MAPK1','MAPK3'))

tmp=read.table('Cell-aging.txt')

Aging=list(tmp$V1)

differentiation=list(c('IL2','NKX2-3','LGALS1','IL10','XBP1','CR1','ITM2A'))

chemotaxis=list(c('GAS6','PTK2B','PIK3CD','XCL1','CH25H','CYP7B1','HSD3B7','CXCL13'))

tmp=read.table('Bcell-activation.txt')

activation=list(tmp$V1)

tmp=read.table('Bcell-proliferation.txt')

proliferation=list(tmp$V1)

subm=readRDS('all.rds')

DefaultAssay(subm)='RNA'

types=c(IFNresponse,Apoptosis,Aging,differentiation,chemotaxis,activation,proliferation)

names=c('IFN response','Apoptosis','Aging','Differentiation','Chemotaxis','Activation','Proliferation')

ps=matrix(nrow=0,ncol=6)

cells=c("PC","B-memory")

for(i in 1:length(cells)){

subc=subset(subm,subType==cells[i])

for(j in 1:length(names)){

subc=AddModuleScore(object = subc, features = types[j],ctrl = 10,name = 'score')

tmp=subc@meta.data[,c(5,7,8,13,14)]

colnames(tmp)=c('sample','seurat_clusters','group','subType','score')

tmp$Type=names[j]

ps=rbind(ps,tmp)

}

}

write.table(ps,file='B-immune.score.txt',sep='\t',row.names = T,col.names = T,quote = F)

ps=read.table('B-immune.score.txt',sep='\t',head=T,row.names=1)

ps1=subset(ps,subType=="PC")

ps2=subset(ps,subType=="B-memory")

pdf('B-immune.score.pdf',width=8,height=5)

my_comparisons=list(c('M','H'),c('S','H'),c('S','M'))

p1=ggboxplot(ps1, x="group", y="score",fill='group', size=0.1,facet.by = "Type",ncol=7,width=0.6, strip.position='top',legend = "none",scales='free_y',outlier.shape = NA,xlab='',ylab='PC')+rotate_x_text(angle = 0)

p1=p1+stat_compare_means(comparisons=my_comparisons,method = "t.test",label = "p.signif",hide.ns = TRUE,vjust=0.5)

p1=p1+scale_fill_manual(values=c('grey','DeepSkyBlue','LightCoral'),name='')

p2=ggboxplot(ps2, x="group", y="score",fill='group', size=0.1,facet.by = "Type",ncol=7,width=0.6, strip.position='top',legend = "none",scales='free_y',outlier.shape = NA,xlab='',ylab='B-memory')+rotate_x_text(angle = 0)

p2=p2+stat_compare_means(comparisons=my_comparisons,method = "t.test",label = "p.signif",hide.ns = TRUE,vjust=0.5)

p2=p2+scale_fill_manual(values=c('grey','DeepSkyBlue','LightCoral'),name='')

p=ggarrange(p1,p2,ncol=1)

print(p)

dev.off()

##Figure 3B

library(psych)

library(ggpubr)

library(reshape2)

library(corrplot)

Inflammation = list(c('IFNG', 'IL10', 'IL12A', 'IL13', 'IL17A', 'IL18','IL1A', 'IL1B', 'IL2', 'IL21', 'IL22', 'IL23A', 'IL4', 'IL5', 'IL6', 'S100A8', 'S100A9','S100A10','S100A11','S100A6','S100A12','TNF','CXCL8'))

IFNresponse =list(c('ADAR','APOBEC3','BST2','CD74','MB21D1','DDIT4','DDX58','DDX60','EIF2AK2','GBP1','GBP2','HPSE','IFI44L','IFI6','IFIH1','IFIT1','IRF1','IRF7','ISG15','ISG20','MAP3K14','MOV10','MS4A4A','MX1','MX2','NAMPT','NT5C3','OAS1','OAS2','OAS3','OASL','P2RY6','PHF15','PML','RSAD2','RTP4','SLC15A3','SLC25A28','SSBP3','TREX1','TRIM5','TRIM25','SUN2','ZC3HAV1','IFITM1','IFITM2','IFITM3'))

MHC = list(c('HLA-DMA','HLA-DMB','HLA-DPA1','HLA-DPB1','HLA-DQA1','HLA-DQB1','HLA-DRA','HLA-DRB1','HLA-DRB5'))

S100= list(c('S100A1','S100A2','S100A3','S100A4','S100A5','S100A6','S100A7','S100A7A','S100A7L2','S100A7P1','S100A7P2','S100A8','S100A9','S100A10','S100A11','S100A12','S100A13','S100A14','S100A15A','S100A16','S100B','S100G','S100P','S100Z'))

Apoptosis= list(c('TNFSF10', 'TNFRSF10A','TNFRSF10B','FASLG','FAS','FADD','TNF','TNFRSF1A','TRADD','CFLAR','CASP8','CASP10','CASP6','CASP3','CASP7','BID','BAX','BAK1','DIABLO','SEPTIN4','HTRA2','CYCS','APAF1','CASP9','PRF1','GZMB','TUBA1B','TUBA4A','TUBA3C','TUBA1A','TUBA1C','TUBA8','TUBA3E','TUBA3D','TUBAL3','MCL1','ACTG1','ACTB','SPTA1','SPTAN1','LMNA','LMNB1','LMNB2','PARP1','PARP2','PARP3','PARP4','DFFA','DFFB','ENDOG','AIFM1','ERN1','TRAF2','ITPR1','ITPR2','ITPR3','CAPN1','CAPN2','CASP12','EIF2AK3','EIF2S1','ATF4','DDIT3','CTSB','CTSC','CTSD','CTSF','CTSH','CTSK','CTSL','CTSO','CTSS','CTSV','CTSW','CTSZ','BIRC2','BIRC3','XIAP','BIRC5','BCL2L11','BCL2L1','BCL2','DAXX','RIPK1','DAB2IP','MAP3K5','MAPK8','MAPK10','MAPK9','BAD','JUN','FOS','TP53','HRK','MAP3K14','CHUK','IKBKB','IKBKG','NFKBIA','NFKB1','RELA','PTPN13','GADD45A','GADD45B','GADD45G','TRAF1','BCL2A1','ATM','PIDD1','TP53AIP1','BBC3','PMAIP1','CASP2','NGF','NTRK1','IL3','IL3RA','CSF2RB','PIK3CA','PIK3CD','PIK3CB','PIK3R1','PIK3R2','PIK3R3','PDPK1','AKT1','AKT2','AKT3','HRAS','KRAS','NRAS','RAF1','MAP2K1','MAP2K2','MAPK1','MAPK3'))

migration =list(c('PECAM1','GAS6','IL4','PLCB1','APP','APOD','PTK2B','HMGB1','TMEM102','GATA3','CCL17','C1QBP','GCSAM','CD99L2','MSMP','ARHGEF5','JAM2','MSTN','TNFSF11','CXCR4','XG','ANXA1','CCL7','CCL8','DUSP1','ZAP70','S100A14','SLC8B1','MAPK1','C5AR1','CKLF','CCL4','PDGFB','PTK2','CCL3L1','CCL22','CMKLR1','CCN3','PIK3CG','TNFRSF14','AKT1','CCL19','KLRK1','MDK','DEFB104A','CXCL17','TNF','EPS8','XCL1','S1PR1','RARRES2','CCL21','SLC12A2','AIF1','FPR2','CXCR2','CXCR1','ITGA4','CCL18','CCL23','ADAM10','CALCA','CCL13','ANO6','CCR2','IL12A','IL34','WNK1','CD99','ARTN','MYO1G','PDGFD','FADD','AKIRIN1','CXCL10','PLA2G7','GREM1','PADI2','CDC42','THBS1','RET','LYN','ICAM1','FLT1','LGALS3','CSF1','CRKL','CRK','CD200R1','TRPM4','KARS1','NBL1','LGALS9','MADCAM1','CCL16','CCL25','RHOA','WASL','GPR15L','JAML','LGMN','IL27RA','CCL24','WNT5A','SPNS2','CD200','CX3CL1','MSN','ITGB7','CXCL11','ALOX5','PIK3CD','SLIT2','STK10','CCR6','CCR5','CCL11','S100A7','AGER','CXCL12','TRPM2','ADAM8','SIRPA','CCL20','ADAM17','SAA1','SERPINE1','TBX21','CCL14','ECM1','RIPOR2','HSD3B7','CCL1','CXCL16','F11R','CD47','PYCARD','IL6R','CCL5','CCL2','S100A12','GPR15','CXCR3','FUT7','CXCL14','CXCL13','AIRE','OXSR1','SPN','ITGAL','SELENOK','CRTAM','CREB3','CCL26','CSF1R','MAPK14','PLG','TNFRSF11A','RPS19','CH25H','DEFB124','CCL4L1','BMP5','SLAMF8','CCL15','XCL2','ADTRP','IL6','CYP7B1','MAPK3','CCL27','RIPK3','MOSPD2','MIA3','GPR183','CCR7','CCR1','STK39','GCSAML','DEFA1','CALR','NLRP12','C3AR1','LRCH1','CCL3','DOCK8','PTPRO'))

tmp=read.table('Cell-aging.txt')

Aging=list(tmp$V1)

types=c(Inflammation,IFNresponse,MHC,S100,Apoptosis,migration,Aging)

names=c('Inflammation','IFNs response','MHC class II','S100 family','Apoptosis','Migration','Aging')

a=read.table('metabolism.term.txt',sep='\t')

b=read.table('KEGG.txt',sep='\t')

colnames(a)=c('ID','Term','Category')

colnames(b)=c('ID','Term','GeneID','Gene')

term=unique(a$ID)

suba=readRDS('all.flt-1.rds')

DefaultAssay(suba)='RNA'

cells=c('Mono-CD14+','Mono-CD16+','Mono-CD14+CD16+')

ps=matrix(ncol=5,nrow=0)

for(i in 1:length(cells)){

subc=subset(suba,subType==cells[i])

for(k in 1:length(term)){

su=subset(b,ID==as.character(term[k]))

for(m in 1:length(names)){

subc=AddModuleScore(object = subc,features = list(su$Gene),name='scoreT',assay = 'RNA',ctrl = 10)

subc=AddModuleScore(object = subc, features = types[m],ctrl = 10,name = 'scoreF')

pv=corr.test(x=subc$scoreT1,y=subc$scoreF1,use='pairwise')

pvd=data.frame(cells[i],term[k],names[m],pv$r,pv$p)

colnames(pvd)=c('cells','ID','DFU','R','P')

ps=rbind(ps,pvd)

}

}

}

psm=merge(ps,a,by='ID',all=TRUE)

write.table(psm,file='metabolic.immune-function.correlation.txt',sep='\t',row.names = F,col.names = T,quote = F)

psm=read.table('metabolic.immune-function.correlation.txt',head=TRUE,sep='\t')

cd14=subset(psm,cells=='Mono-CD14+')

datam=dcast(cd14, DFU ~ Term, value.var='R')

rownames(datam)=datam$DFU

col3 <- colorRampPalette(c("blue","navy","navy","white","darkred","darkred","orange"))

pdf('CD14.metabolic.immune-function.correlation.pdf',width=6,height=6)

corrplot(t(datam[,unique(psm$Term)]),method='circle',is.corr=TRUE,cl.lim=c(-0.55,0.65),tl.cex=0.5,col = col3(1000),tl.srt = 45,tl.col='black',cl.ratio=0.08,cl.align.text='c',cl.pos='b')

dev.off()

cd16=subset(psm,cells=='Mono-CD16+')

datam=dcast(cd16, DFU ~ Term, value.var='R')

rownames(datam)=datam$DFU

datam[is.na(datam)]<-0

col3 <- colorRampPalette(c("blue","navy","navy","white","darkred","darkred","orange"))

pdf('CD16.metabolic.immune-function.correlation.pdf',width=6,height=6)

corrplot(t(datam[,unique(psm$Term)]),method='circle',is.corr=TRUE,cl.lim=c(-0.5,0.74),tl.cex=0.5,col = col3(1000),tl.srt = 45,tl.col='black',cl.ratio=0.08,cl.align.text='c',cl.pos='b')

dev.off()

doup=subset(psm,cells=='Mono-CD14+CD16+')

datam=dcast(doup, DFU ~ Term, value.var='R')

rownames(datam)=datam$DFU

datam[is.na(datam)]<-0

col3 <- colorRampPalette(c("blue","navy","navy","white","darkred","darkred","orange"))

pdf('CD14CD16.metabolic.immune-function.correlation.pdf',width=6,height=6)

corrplot(t(datam[,unique(psm$Term)]),method='circle',is.corr=TRUE,cl.lim=c(-0.5,0.53),tl.cex=0.5,col = col3(1000),tl.srt = 45,tl.col='black',cl.ratio=0.08,cl.align.text='c',cl.pos='b')

dev.off()

##Figure S2B

Apoptosis= list(c('TNFSF10', 'TNFRSF10A','TNFRSF10B','FASLG','FAS','FADD','TNF','TNFRSF1A','TRADD','CFLAR','CASP8','CASP10','CASP6','CASP3','CASP7','BID','BAX','BAK1','DIABLO','SEPTIN4','HTRA2','CYCS','APAF1','CASP9','PRF1','GZMB','TUBA1B','TUBA4A','TUBA3C','TUBA1A','TUBA1C','TUBA8','TUBA3E','TUBA3D','TUBAL3','MCL1','ACTG1','ACTB','SPTA1','SPTAN1','LMNA','LMNB1','LMNB2','PARP1','PARP2','PARP3','PARP4','DFFA','DFFB','ENDOG','AIFM1','ERN1','TRAF2','ITPR1','ITPR2','ITPR3','CAPN1','CAPN2','CASP12','EIF2AK3','EIF2S1','ATF4','DDIT3','CTSB','CTSC','CTSD','CTSF','CTSH','CTSK','CTSL','CTSO','CTSS','CTSV','CTSW','CTSZ','BIRC2','BIRC3','XIAP','BIRC5','BCL2L11','BCL2L1','BCL2','DAXX','RIPK1','DAB2IP','MAP3K5','MAPK8','MAPK10','MAPK9','BAD','JUN','FOS','TP53','HRK','MAP3K14','CHUK','IKBKB','IKBKG','NFKBIA','NFKB1','RELA','PTPN13','GADD45A','GADD45B','GADD45G','TRAF1','BCL2A1','ATM','PIDD1','TP53AIP1','BBC3','PMAIP1','CASP2','NGF','NTRK1','IL3','IL3RA','CSF2RB','PIK3CA','PIK3CD','PIK3CB','PIK3R1','PIK3R2','PIK3R3','PDPK1','AKT1','AKT2','AKT3','HRAS','KRAS','NRAS','RAF1','MAP2K1','MAP2K2','MAPK1','MAPK3'))

Cytotoxicity=list(c('PRF1', 'IFNG', 'GNLY', 'NKG7', 'GZMB', 'GZMA', 'GZMH', 'KLRK1', 'KLRB1', 'KLRD1', 'CTSW', 'CST7'))

Exhaustion=list(c('LAG3', 'TIGIT', 'PDCD1', 'CTLA4', 'HAVCR2', 'TOX'))

migration =list(c('APP','APOD','TMEM102','CD99L2','XG','ZAP70','PIK3CG','TNFRSF14','XCL1','S1PR1','CCL21','SLC12A2','AIF1','ITGA4','ADAM10','CCR2','WNK1','CD99','MYO1G','FADD','CXCL10','ICAM1','CRKL','CRK','CD200R1','RHOA','IL27RA','WNT5A','CD200','MSN','ITGB7','CXCL11','PIK3CD','CCR6','S100A7','CXCL12','ADAM8','CCL20','ADAM17','RIPOR2','CXCL16','F11R','PYCARD','CCL5','CCL2','GPR15','CXCR3','CXCL13','AIRE','OXSR1','SPN','ITGAL','SELENOK','CCL26','XCL2','CCL27','RIPK3','GPR183','STK39','DEFA1','LRCH1','CCL3','DOCK8'))

IFNresponse =list(c('ADAR','APOBEC3','BST2','CD74','MB21D1','DDIT4','DDX58','DDX60','EIF2AK2','GBP1','GBP2','HPSE','IFI44L','IFI6','IFIH1','IFIT1','IRF1','IRF7','ISG15','ISG20','MAP3K14','MOV10','MS4A4A','MX1','MX2','NAMPT','NT5C3','OAS1','OAS2','OAS3','OASL','P2RY6','PHF15','PML','RSAD2','RTP4','SLC15A3','SLC25A28','SSBP3','TREX1','TRIM5','TRIM25','SUN2','ZC3HAV1','IFITM1','IFITM2','IFITM3'))

tmp=read.table('T-cell-activation.txt')

Act=list(tmp$V1)

tmp=read.table('T-cell-differentiation.txt')

Dif=list(tmp$V1)

tmp=read.table('T-cell-proliferation.txt')

Pro=list(tmp$V1)

tmp=read.table('Cell-aging.txt')

Aging=list(tmp$V1)

types=c(Act,Aging,Apoptosis,Dif,IFNresponse,Pro)

names=c('Activation','Aging','Apoptosis','Differentiation','IFNs response','Proliferation')

a=read.table('metabolism.term.txt',sep='\t')

b=read.table('KEGG.txt',sep='\t')

colnames(a)=c('ID','Term','Category')

colnames(b)=c('ID','Term','GeneID','Gene')

term=unique(a$ID)

suba=readRDS('all.rds')

DefaultAssay(suba)='RNA'

cells=c('CD4-FOXP3','CD4-ICOS','CD8-CCR7','CD8-GZMB')

ps=matrix(ncol=5,nrow=0)

for(i in 1:length(cells)){

subc=subset(suba,subType==cells[i])

for(k in 1:length(term)){

su=subset(b,ID==as.character(term[k]))

for(m in 1:length(names)){

subc=AddModuleScore(object = subc,features = list(su$Gene),name='scoreT',assay = 'RNA',ctrl = 10)

subc=AddModuleScore(object = subc, features = types[m],ctrl = 10,name = 'scoreF')

pv=corr.test(x=subc$scoreT1,y=subc$scoreF1,use='pairwise')

pvd=data.frame(cells[i],term[k],names[m],pv$r,pv$p)

colnames(pvd)=c('cells','ID','DFU','R','P')

ps=rbind(ps,pvd)

}

}

}

psm=merge(ps,a,by='ID',all=TRUE)

write.table(psm,file='T-metabolic.immune-function.correlation.txt',sep='\t',row.names = F,col.names = T,quote = F)

psm=read.table('T-metabolic.immune-function.correlation.txt',head=TRUE,sep='\t')

c7=subset(psm,cells=='CD4-FOXP3')

datam=dcast(c7, DFU ~ Term, value.var='R')

rownames(datam)=datam$DFU

col3 <- colorRampPalette(c("blue","navy","navy","white","darkred","darkred","orange"))

pdf('CD4-FOXP3.metabolic.immune-function.correlation.pdf',width=9,height=6)

corrplot(t(datam[,unique(psm$Term)]),method='circle',is.corr=TRUE,cl.lim=c(-0.5,0.45),tl.cex=0.5,col = col3(1000),tl.srt = 45,tl.col='black',cl.ratio=0.08,cl.align.text='c',cl.pos='b')

dev.off()

c3=subset(psm,cells=='CD4-ICOS')

datam=dcast(c3, DFU ~ Term, value.var='R')

rownames(datam)=datam$DFU

datam[is.na(datam)]<-0

col3 <- colorRampPalette(c("blue","navy","navy","white","darkred","darkred","orange"))

pdf('CD4-ICOS.metabolic.immune-function.correlation.pdf',width=9,height=6)

corrplot(t(datam[,unique(psm$Term)]),method='circle',is.corr=TRUE,cl.lim=c(-0.52,0.5),tl.cex=0.5,col = col3(1000),tl.srt = 45,tl.col='black',cl.ratio=0.08,cl.align.text='c',cl.pos='b')

dev.off()

cg=subset(psm,cells=='CD8-GZMB')

datam=dcast(cg, DFU ~ Term, value.var='R')

rownames(datam)=datam$DFU

datam[is.na(datam)]<-0

col3 <- colorRampPalette(c("blue","navy","navy","white","darkred","darkred","orange"))

pdf('CD8-GZMB.metabolic.immune-function.correlation.pdf',width=9,height=6)

corrplot(t(datam[,unique(psm$Term)]),method='circle',is.corr=TRUE,cl.lim=c(-0.5,0.40),tl.cex=0.5,col = col3(1000),tl.srt = 45,tl.col='black',cl.ratio=0.08,cl.align.text='c',cl.pos='b')

dev.off()

cg=subset(psm,cells=='CD8-CCR7')

datam=dcast(cg, DFU ~ Term, value.var='R')

rownames(datam)=datam$DFU

datam[is.na(datam)]<-0

col3 <- colorRampPalette(c("blue","navy","navy","white","darkred","darkred","orange"))

pdf('CD8-CCR7.metabolic.immune-function.correlation.pdf',width=9,height=6)

corrplot(t(datam[,unique(psm$Term)]),method='circle',is.corr=TRUE,cl.lim=c(-0.4,0.40),tl.cex=0.5,col = col3(1000),tl.srt = 45,tl.col='black',cl.ratio=0.08,cl.align.text='c',cl.pos='b')

dev.off()

##Figure 4B

IFNresponse =list(c('ADAR','APOBEC3','BST2','CD74','MB21D1','DDIT4','DDX58','DDX60','EIF2AK2','GBP1','GBP2','HPSE','IFI44L','IFI6','IFIH1','IFIT1','IRF1','IRF7','ISG15','ISG20','MAP3K14','MOV10','MS4A4A','MX1','MX2','NAMPT','NT5C3','OAS1','OAS2','OAS3','OASL','P2RY6','PHF15','PML','RSAD2','RTP4','SLC15A3','SLC25A28','SSBP3','TREX1','TRIM5','TRIM25','SUN2','ZC3HAV1','IFITM1','IFITM2','IFITM3'))

Apoptosis= list(c('TNFSF10', 'TNFRSF10A','TNFRSF10B','FASLG','FAS','FADD','TNF','TNFRSF1A','TRADD','CFLAR','CASP8','CASP10','CASP6','CASP3','CASP7','BID','BAX','BAK1','DIABLO','SEPTIN4','HTRA2','CYCS','APAF1','CASP9','PRF1','GZMB','TUBA1B','TUBA4A','TUBA3C','TUBA1A','TUBA1C','TUBA8','TUBA3E','TUBA3D','TUBAL3','MCL1','ACTG1','ACTB','SPTA1','SPTAN1','LMNA','LMNB1','LMNB2','PARP1','PARP2','PARP3','PARP4','DFFA','DFFB','ENDOG','AIFM1','ERN1','TRAF2','ITPR1','ITPR2','ITPR3','CAPN1','CAPN2','CASP12','EIF2AK3','EIF2S1','ATF4','DDIT3','CTSB','CTSC','CTSD','CTSF','CTSH','CTSK','CTSL','CTSO','CTSS','CTSV','CTSW','CTSZ','BIRC2','BIRC3','XIAP','BIRC5','BCL2L11','BCL2L1','BCL2','DAXX','RIPK1','DAB2IP','MAP3K5','MAPK8','MAPK10','MAPK9','BAD','JUN','FOS','TP53','HRK','MAP3K14','CHUK','IKBKB','IKBKG','NFKBIA','NFKB1','RELA','PTPN13','GADD45A','GADD45B','GADD45G','TRAF1','BCL2A1','ATM','PIDD1','TP53AIP1','BBC3','PMAIP1','CASP2','NGF','NTRK1','IL3','IL3RA','CSF2RB','PIK3CA','PIK3CD','PIK3CB','PIK3R1','PIK3R2','PIK3R3','PDPK1','AKT1','AKT2','AKT3','HRAS','KRAS','NRAS','RAF1','MAP2K1','MAP2K2','MAPK1','MAPK3'))

tmp=read.table('Cell-aging.txt')

Aging=list(tmp$V1)

differentiation=list(c('IL2','NKX2-3','LGALS1','IL10','XBP1','CR1','ITM2A'))

chemotaxis=list(c('GAS6','PTK2B','PIK3CD','XCL1','CH25H','CYP7B1','HSD3B7','CXCL13'))

tmp=read.table('Bcell-activation.txt')

activation=list(tmp$V1)

tmp=read.table('Bcell-proliferation.txt')

proliferation=list(tmp$V1)

types=c(IFNresponse,Apoptosis,Aging,differentiation,chemotaxis,activation,proliferation)

names=c('IFN response','Apoptosis','Aging','Differentiation','Chemotaxis','Activation','Proliferation')

a=read.table('metabolism.term.txt',sep='\t')

b=read.table('KEGG.txt',sep='\t')

colnames(a)=c('ID','Term','Category')

colnames(b)=c('ID','Term','GeneID','Gene')

term=unique(a$ID)

suba=readRDS('all.rds')

DefaultAssay(suba)='RNA'

cells=c("PC","B-memory")

ps=matrix(ncol=5,nrow=0)

for(i in 1:length(cells)){

subc=subset(suba,subType==cells[i])

for(k in 1:length(term)){

su=subset(b,ID==as.character(term[k]))

for(m in 1:length(names)){

subc=AddModuleScore(object = subc,features = list(su$Gene),name='scoreT',assay = 'RNA',ctrl = 10)

subc=AddModuleScore(object = subc, features = types[m],ctrl = 10,name = 'scoreF')

pv=corr.test(x=subc$scoreT1,y=subc$scoreF1,use='pairwise')

pvd=data.frame(cells[i],term[k],names[m],pv$r,pv$p)

colnames(pvd)=c('cells','ID','DFU','R','P')

ps=rbind(ps,pvd)

}

}

}

psm=merge(ps,a,by='ID',all=TRUE)

write.table(psm,file='B-metabolic.immune-function.correlation.txt',sep='\t',row.names = F,col.names = T,quote = F)

psm=read.table('B-metabolic.immune-function.correlation.txt',head=TRUE,sep='\t')

c7=subset(psm,cells=='PC')

datam=dcast(c7, DFU ~ Term, value.var='R')

rownames(datam)=datam$DFU

col3 <- colorRampPalette(c("blue","navy","navy","white","darkred","darkred","orange"))

pdf('PC.metabolic.immune-function.correlation.pdf',width=9,height=6)

corrplot(t(datam[,as.character(a$Term)]),method='circle',is.corr=TRUE,cl.lim=c(-0.34,0.5),tl.cex=0.5,col = col3(1000),tl.srt = 45,tl.col='black',cl.ratio=0.08,cl.align.text='c',cl.pos='b')

dev.off()

cg=subset(psm,cells=='B-memory')

datam=dcast(cg, DFU ~ Term, value.var='R')

rownames(datam)=datam$DFU

datam[is.na(datam)]<-0

col3 <- colorRampPalette(c("blue","navy","navy","white","darkred","darkred","orange"))

pdf('B-memory.metabolic.immune-function.correlation.pdf',width=9,height=6)

corrplot(t(datam[,as.character(a$Term)]),method='circle',is.corr=TRUE,cl.lim=c(-0.5,0.44),tl.cex=0.5,col = col3(1000),tl.srt = 45,tl.col='black',cl.ratio=0.08,cl.align.text='c',cl.pos='b')

dev.off()

##Figure 3C and Figure S1

library(psych)

library(corrplot)

subc=readRDS('all.rds')

degs=read.table('all.DEGs.txt',sep='\t',head=T)

types=c('Mono-CD14+','Mono-CD16+','Mono-CD14+CD16+','CD4-FOXP3','CD4-ICOS','CD8-CCR7','CD8-GZMB','B-memory','PC')

for(j in 1:length(types)){

deg=subset(degs,CellType==types[j])

subt=subset(subc,subType==types[j])

genes=as.character(unique(deg$Gene))

genes=genes[!grepl("MT-|^RP[SL]",genes)]

a0=read.table('../../metabolism.term.txt',sep='\t')

b0=read.table('../../KEGG.txt',sep='\t')

b0=subset(b0,V1 %in% a0$V1)

d0=subset(b0,V4 %in% genes)

colnames(d0)=c('ID','Term','geneID','geneName')

write.table(d0,file=paste(types[j],'.DEGs.metabolism.txt',sep=''),sep='\t',row.names=F,col.names=T,quote=F)

gr=c('H','M','S')

for(k in 1:3){

subm=subset(subt,group==as.character(gr[k]))

n=dim(subm[['RNA']]@data)[1]

chunk <- function(x,n) split(x, factor(sort(rank(x)%%n)))

spar=chunk(colnames(subm),100)

input=matrix(nrow=n,ncol=0)

for(e in 1:length(spar)){

cell=spar[[e]]

data=subm[['RNA']]@data[,cell]

average_data=data.frame(apply(data,1,mean))

colnames(average_data)=paste('N',e,sep='')

input=cbind(input,average_data)

}

inputs=input[as.character(genes),]

write.table(inputs,file=paste(types[j],'.',gr[k],'.matrix.txt',sep=''),sep='\t',row.names=T,col.names=T,quote=F)

}

}

cov=t(as.matrix(read.table('Mono-CD14+.S.matrix.txt',head=T,sep='\t')))

covcor1=corr.test(cov)

cov=t(as.matrix(read.table('Mono-CD14+.M.matrix.txt',head=T,sep='\t')))

covcor2=corr.test(cov)

cov=t(as.matrix(read.table('Mono-CD14+.H.matrix.txt',head=T,sep='\t')))

covcor3=corr.test(cov)

pdf('Mono-CD14+.co-expression.pdf',width=10,height=10)

p1=corrplot(covcor1$r,method='color',type='upper',is.corr=TRUE,order='hclust',hclust.method=c('complete'),addrect = 5,col=colorRampPalette(c('blue4', 'blue', 'white', 'orange', 'red3'))(100),tl.pos='lt',tl.srt = 90,tl.col='black',tl.cex=0.4,pch.cex=1,diag = TRUE)

write.table(rownames(p1),file='Mono-CD14+.order.txt',sep='\t',row.names=F,col.names=T,quote=F)

p2=corrplot(covcor2$r[rownames(p1),colnames(p1)],add = TRUE,method='color',type='lower',is.corr=TRUE,col=colorRampPalette(c('blue4', 'blue', 'white', 'orange', 'red3'))(100),diag = TRUE, tl.pos = 'n',tl.col='black',tl.cex=0.4, cl.pos = "n")

p3=corrplot(covcor3$r[rownames(p1),colnames(p1)],method='color',type='upper',is.corr=TRUE,col=colorRampPalette(c('blue4', 'blue', 'white', 'orange', 'red3'))(100),tl.pos='lt',tl.srt = 90,tl.col='black',tl.cex=0.4,pch.cex=1,diag = TRUE)

dev.off()

cov=t(as.matrix(read.table('Mono-CD16+.S.matrix.txt',head=T,sep='\t')))

cov=cov[,c(1:203,205:1242)]

# delete AC104809.4

covcor1=corr.test(cov)

cov=t(as.matrix(read.table('Mono-CD16+.M.matrix.txt',head=T,sep='\t')))

covcor2=corr.test(cov)

cov=t(as.matrix(read.table('Mono-CD16+.H.matrix.txt',head=T,sep='\t')))

covcor3=corr.test(cov)

pdf('Mono-CD16+.co-expression-1.pdf',width=10,height=10)

p1=corrplot(covcor1$r,method='color',type='upper',is.corr=TRUE,order='hclust',hclust.method=c('complete'),addrect = 5,col=colorRampPalette(c('blue4', 'blue', 'white', 'orange', 'red3'))(100),tl.pos='n',tl.srt = 90,tl.col='black',tl.cex=0.1,pch.cex=0,diag = TRUE)

write.table(rownames(p1),file='Mono-CD16+.order.txt',sep='\t',row.names=F,col.names=T,quote=F)

p2=corrplot(covcor2$r[rownames(p1),colnames(p1)],add = TRUE,method='color',type='lower',is.corr=TRUE,col=colorRampPalette(c('blue4', 'blue', 'white', 'orange', 'red3'))(100),diag = TRUE, tl.pos = 'n',tl.col='black',tl.cex=0.1, cl.pos = "n")

p3=corrplot(covcor3$r[rownames(p1),colnames(p1)],method='color',type='upper',is.corr=TRUE,col=colorRampPalette(c('blue4', 'blue', 'white', 'orange', 'red3'))(100),tl.pos='n',tl.srt = 90,tl.col='black',tl.cex=0.1,pch.cex=0,diag = TRUE)

dev.off()

cov=t(as.matrix(read.table('Mono-CD14+CD16+.S.matrix.txt',head=T,sep='\t')))

covcor1=corr.test(cov)

cov=t(as.matrix(read.table('Mono-CD14+CD16+.M.matrix.txt',head=T,sep='\t')))

covcor2=corr.test(cov)

cov=t(as.matrix(read.table('Mono-CD14+CD16+.H.matrix.txt',head=T,sep='\t')))

covcor3=corr.test(cov)

pdf('Mono-CD14+CD16+.co-expression.pdf',width=10,height=10)

p1=corrplot(covcor1$r,method='color',type='upper',is.corr=TRUE,order='hclust',hclust.method=c('complete'),addrect = 5,col=colorRampPalette(c('blue4', 'blue', 'white', 'orange', 'red3'))(100),tl.pos='lt',tl.srt = 90,tl.col='black',tl.cex=0.4,pch.cex=1,diag = TRUE)

write.table(rownames(p1),file='Mono-CD14+CD16+.order.txt',sep='\t',row.names=F,col.names=T,quote=F)

p2=corrplot(covcor2$r[rownames(p1),colnames(p1)],add = TRUE,method='color',type='lower',is.corr=TRUE,col=colorRampPalette(c('blue4', 'blue', 'white', 'orange', 'red3'))(100),diag = TRUE, tl.pos = 'n',tl.col='black',tl.cex=0.4, cl.pos = "n")

p3=corrplot(covcor3$r[rownames(p1),colnames(p1)],method='color',type='upper',is.corr=TRUE,col=colorRampPalette(c('blue4', 'blue', 'white', 'orange', 'red3'))(100),tl.pos='lt',tl.srt = 90,tl.col='black',tl.cex=0.4,pch.cex=1,diag = TRUE)

dev.off()

##Figure 4C and Figure 4D

cov=t(as.matrix(read.table('PC.S.matrix.txt',head=T,sep='\t')))

covcor1=corr.test(cov)

cov=t(as.matrix(read.table('PC.M.matrix.txt',head=T,sep='\t')))

covcor2=corr.test(cov)

pdf('PC.co-expression.pdf',width=8,height=8)

p1=corrplot(covcor1$r,method='color',type='upper',is.corr=TRUE,order='hclust',hclust.method=c('complete'),addrect = 5,col=colorRampPalette(c('blue4', 'blue', 'white', 'orange', 'red3'))(100),tl.pos='lt',tl.srt = 90,tl.col='black',tl.cex=0.4,pch.cex=1,diag = TRUE)

write.table(rownames(p1),file='PC.order.txt',sep='\t',row.names=F,col.names=T,quote=F)

p2=corrplot(covcor2$r[rownames(p1),colnames(p1)],add = TRUE,method='color',type='lower',is.corr=TRUE,col=colorRampPalette(c('blue4', 'blue', 'white', 'orange', 'red3'))(100),diag = TRUE, tl.pos = 'n',tl.col='black',tl.cex=0.4, cl.pos = "n")

dev.off()

cov=t(as.matrix(read.table('B-memory.S.matrix.txt',head=T,sep='\t')))

covcor1=corr.test(cov)

cov=t(as.matrix(read.table('B-memory.M.matrix.txt',head=T,sep='\t')))

covcor2=corr.test(cov)

cov=t(as.matrix(read.table('B-memory.H.matrix.txt',head=T,sep='\t')))

covcor3=corr.test(cov)

pdf('B-memory.co-expression.pdf',width=7,height=7)

p1=corrplot(covcor1$r,method='color',type='upper',is.corr=TRUE,order='hclust',hclust.method=c('complete'),addrect = 5,col=colorRampPalette(c('blue4', 'blue', 'white', 'orange', 'red3'))(100),tl.pos='lt',tl.srt = 90,tl.col='black',tl.cex=0.4,pch.cex=1,diag = TRUE)

write.table(rownames(p1),file='B-memory.order.txt',sep='\t',row.names=F,col.names=T,quote=F)

p2=corrplot(covcor2$r[rownames(p1),colnames(p1)],add = TRUE,method='color',type='lower',is.corr=TRUE,col=colorRampPalette(c('blue4', 'blue', 'white', 'orange', 'red3'))(100),diag = TRUE, tl.pos = 'n',tl.col='black',tl.cex=0.4, cl.pos = "n")

p3=corrplot(covcor3$r[rownames(p1),colnames(p1)],method='color',type='upper',is.corr=TRUE,col=colorRampPalette(c('blue4', 'blue', 'white', 'orange', 'red3'))(100),tl.pos='lt',tl.srt = 90,tl.col='black',tl.cex=0.4,pch.cex=1,diag = TRUE)

dev.off()
